# Continuous‐flow Synthesis of Aryl Aldehydes by Pd‐catalyzed Formylation of Aryl Bromides Using Carbon Monoxide and Hydrogen

**DOI:** 10.1002/cssc.201802261

**Published:** 2018-11-13

**Authors:** Christopher A. Hone, Pavol Lopatka, Rachel Munday, Anne O'Kearney‐McMullan, C. Oliver Kappe

**Affiliations:** ^1^ Center for Continuous Flow Synthesis and Processing (CCFLOW) Research Center Pharmaceutical Engineering (RCPE) Inffeldgasse 13 8010 Graz Austria; ^2^ Institute of Chemistry University of Graz, NAWI Graz Heinrichstrasse 28 A-8010 Graz Austria; ^3^ AstraZeneca Silk Road Business Park Macclesfield SK10 2NA UK

**Keywords:** aldehydes, carbonylation, continuous-flow, homogeneous catalysis, palladium

## Abstract

A continuous‐flow protocol utilizing syngas (CO and H_2_) was developed for the palladium‐catalyzed reductive carbonylation of (hetero)aryl bromides to their corresponding (hetero)aryl aldehydes. The optimization of temperature, pressure, catalyst and ligand loading, and residence time resulted in process‐intensified flow conditions for the transformation. In addition, a key benefit of investigating the reaction in flow is the ability to precisely control the CO‐to‐H_2_ stoichiometric ratio, which was identified as having a critical influence on yield. The protocol proceeds with low catalyst and ligand loadings: palladium acetate (1 mol % or below) and cata*CX*ium A (3 mol % or below). A variety of (hetero)aryl bromides at a 3 mmol scale were converted to their corresponding (hetero)aryl aldehydes at 12 bar pressure (CO/H_2_=1:3) and 120 °C reaction temperature within 45 min residence time to afford products mostly in good‐to‐excellent yields (17 examples). In particular, a successful scale‐up was achieved over 415 min operation time for the reductive carbonylation of 2‐bromo‐6‐methoxynaphthalene to synthesize 3.8 g of 6‐methoxy‐2‐naphthaldehyde in 85 % isolated yield. Studies were conducted to understand catalyst decomposition within the reactor by using inductively coupled plasma–mass spectrometry (ICP–MS) analysis. The palladium could easily be recovered using an aqueous nitric acid wash post reaction. Mechanistic aspects and the scope of the transformation are discussed.

## Introduction

**Figure 1:**
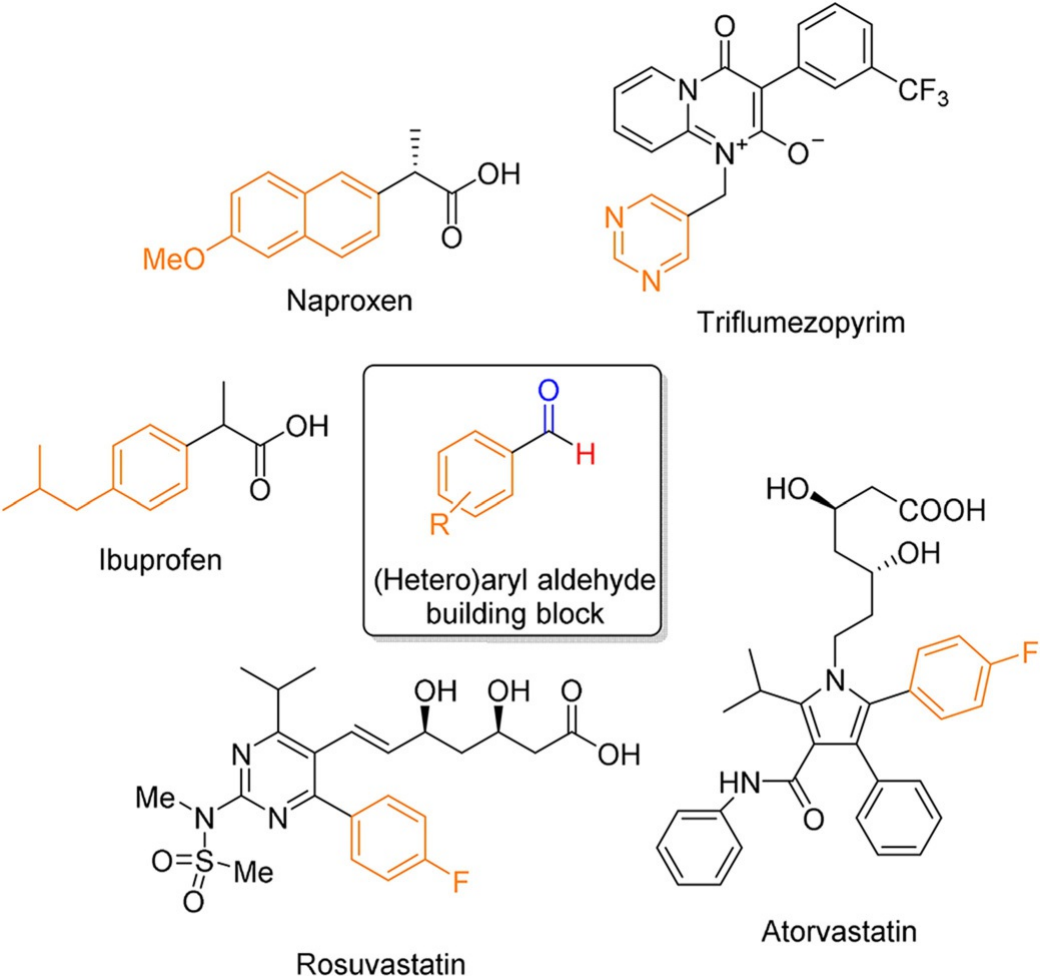
Important biologically active molecules containing building blocks derived from (hetero)aryl aldehydes.

**Scheme 1:**
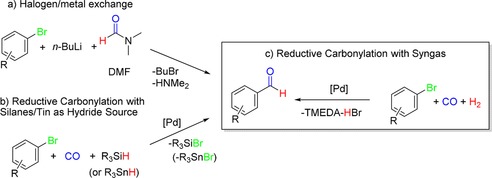
Synthetic approaches for the formation of aryl aldehydes from aryl bromides.

Aryl and heteroaryl aldehydes are important intermediates in the synthesis of biologically active molecules (Figure 1). There are a number of synthetic strategies to form aryl aldehydes from their corresponding aryl bromides. One strategy is to use halogen–lithium exchange and subsequently react the lithium intermediate with dimethylformamide (DMF) (Scheme 1 a).^1^ However, this protocol requires stoichiometric amounts of metal and has limited substrate scope due to the sensitivity of some substrates to decomposition by a strong base such as *n*‐butyllithium (*n*BuLi). Pd‐catalyzed formylation of aryl bromides has emerged as a powerful methodology in the organic synthesis of carbonyl compounds.^2^ In the case of reductive carbonylations, CO is used in combination with a hydrogen donor source, such as silyl and tin hydrides, or formate salts, to achieve formylation at low pressure (Scheme 1 b).^3^, ^4^ However, these protocols often require high catalyst loadings, and silicon and tin hydrides are relatively expensive, which limits their use. In particular, Sn is highly undesirable for pharmaceutical manufacture due to contamination and toxicity. In addition, in the case of silyl hydrides, the silyl hydride is typically added in excess (≈2–3 equiv), which increases costs and complicates post‐reaction processing.

Anastas and Kirchhoff developed the 12 principles of green chemistry as a response for the necessity to reduce the environmental impact of chemicals.^5^ Perhaps the greenest and most atom‐economic source of CO and H_2_ is synthesis gas (syngas, CO/H_2_). Syngas is a highly abundant and inexpensive feedstock that is available from many sources in the chemicals industry.^6^ Syngas can be produced from the splitting of water and activation of carbon dioxide by electrolysis. Furthermore, virtually all hydrocarbons, derived from natural gas, petroleum, and coal, can be used as a feedstock for the production of syngas through partial oxidation, steam reforming, or gasification. Future sustainable energy policies are likely to see an increase in the use of biomass or municipal waste for syngas production. The first palladium‐catalyzed formylation utilizing syngas was reported in 1974 by Schoenberg and Heck using [Pd(PPh_3_)_2_Cl_2_] as catalyst at very high pressures (80–100 bar) and elevated temperatures (80–150 °C).^7^ The protocol was not widely adopted for conventional organic synthesis due to the high pressures required. In 2006, Beller and co‐workers reported the formylation of aryl and heteroaryl bromides by Pd(OAc)_2_, di(1‐adamantyl)‐*n*‐butylphosphine (cata*CX*ium A), tetramethylethylenediamine (TMEDA) as base, using synthesis gas (CO/H_2_=1:1) at relatively low pressures (5–7 bar) with 16 h reaction time (Scheme 1 c).^8^ The protocol utilized very low loadings of catalyst (0.25 mol %) and ligand (0.75 mol %) in most cases and was demonstrated on a wide substrate scope.

There are many process challenges associated with handling gas–liquid transformations in batch reactors, particularly at large scales. The interfacial area between the gas and liquid phases becomes proportionally smaller with increasing reactor size; therefore, the reaction is more likely to be mass transfer limited, which leads to reproducibility problems during scale up. In addition, most of the gas is in the headspace and therefore the reactor needs to be pressurized to maximize the amount of gas in solution and reduce mass transfer effects. A large inventory of highly poisonous CO and extremely flammable H_2_ needs to be loaded and pressurized into the batch vessel from a gas cylinder. Typical commercial batch reactors can operate between 2 and 6 bar; these higher pressures require more specialized and expensive equipment. These challenges and that the reaction utilizes toxic and flammable gases unfortunately renders this transformation increasingly unacceptable in contemporary organic synthesis within a batch environment. One solution is to form the gas or gases in situ or ex situ from solid or liquid reagents (gas surrogate) that liberate CO/H_2_, which addresses some of the challenges associated with handling gases. The problem is that in situ formation generally requires the presence of a transition‐metal catalyst and strong base in combination with high temperatures (>100 °C) to release CO, therefore often resulting in compatibility issues between the CO‐producing and CO‐consuming reaction.^9^ Pioneering research by the Skrydstrup group resulted in the development of a two‐chamber batch solution for forming gases ex situ.^10^ In particular, 9‐methylfluorene‐9‐carbonyl chloride was employed to generate stoichiometric amounts of CO and potassium formate as the hydride source within a two‐chamber system (COware) for reductive carbonylation of aryl iodides.^11^ Madsen and co‐workers also demonstrated a two batch chamber configuration for the ex situ formation of CO and H_2_ using an iridium‐catalyzed dehydrogenative decarbonylation of hexane‐1,6‐diol, which was fed into a second chamber for the formylation of aryl bromides.^12^ The Ley group pioneered the tube‐in‐tube reactor gas‐loading concept to enable the safer introduction of gases into the liquid‐phase from gas cylinders.^13^ Teflon AF‐2400 (a fluoropolymer) is used as a semipermeable membrane, which is permeable to gases but impermeable to liquids. The tube‐in‐tube flow reactor was successfully applied for hydroformylation and some carbonylation reactions but not specifically for reductive carbonylation reactions.^14^ Furthermore, Ley and co‐workers recently showed that oxalyl chloride can be hydrolyzed by using NaOH to form CO in situ in flow and subsequently used the generated CO in carbonylation reactions.^15^ The aforementioned strategies are good options for research‐scale experimentation; however, both approaches suffer from limited scalability in terms of atom inefficiency, poorer performance at scale‐up, or are simply too expensive.^16^, ^17^

Tubular plug flow reactors have emerged as a platform for the safe, efficient, and scalable utilization of gases direct from gas cylinders by using mass‐flow controllers.^17^ Gas–liquid reactions have successfully been applied for the synthesis of active pharmaceutical intermediates (APIs) by using continuous‐flow reactors.^18^ The improved safety features of continuous‐flow reactors enable the safe operation at higher pressures and temperatures, including above the boiling point of the solvent.^19^ A continuous‐flow reactor only needs a relatively small pressurized reactor volume containing the reaction mixture and, when properly designed, can sustain the pressure of an unexpected combustion.^20^ Gas–liquid segmented (Taylor) flow regimes generated in flow microchannels provide a high interfacial area between the gas and liquid phases within a tubular flow reactor, therefore mass‐transfer effects are minimized. The utilization of CO in flow for organic synthesis has been demonstrated by a number of groups.^21^ In particular, Ryu and co‐workers demonstrated a microflow process for a radical‐based carbonylation reaction of alkyl iodides and bromides to aldehydes and ketones.^22^ However, this procedure required environmentally unfriendly and expensive Bu_3_SnH and very high CO pressures (80 bar). At Eli Lilly, the successful and safe scale‐up of hydroformylation and reductive amination was demonstrated by using CO and H_2_ within a large‐scale tubular reactor, with one example demonstrated at a 2 MT scale.^23^, ^24^, ^25^

We were inspired by the low pressure batch protocol reported by Beller and co‐workers for the reductive carbonylation of (hetero)aryl bromides with synthesis gas as a sustainable and cost effective reagent.^8^ We herein report the development of a continuous‐flow protocol for the reductive carbonylation of (hetero)aryl bromides to (hetero)aryl aldehydes using synthesis gas. To our knowledge, this is the first reported flow procedure for the reductive carbonylation of (hetero)aryl bromides using syngas.

## Results and Discussion

**Figure 2:**
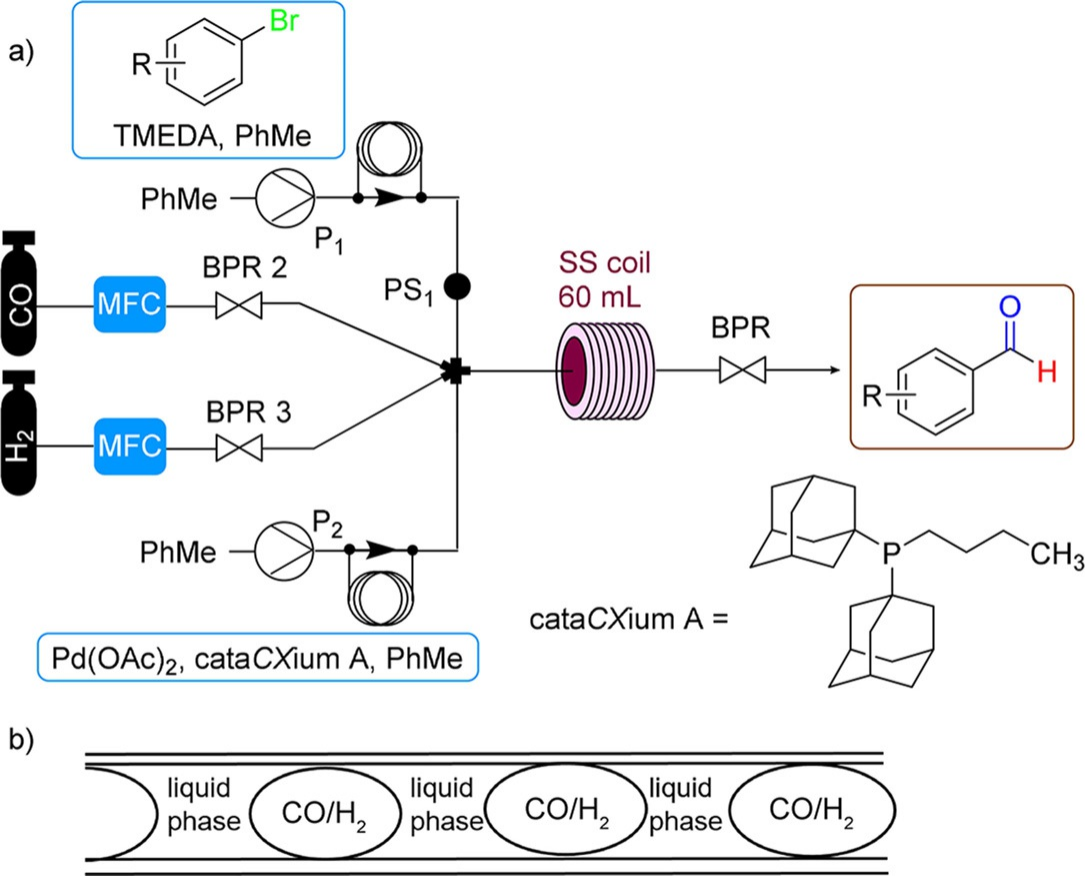
(a) Continuous‐flow configuration for reductive carbonylation optimization; (b) gas–liquid segmented (Taylor) flow regime.

The gas–liquid continuous‐flow reactor setup consisted of two high‐pressure liquid pumps (HPLC) (P, Uniqsis) for introducing the liquid feeds (see Figure 2 a; see also Figure S1 in the Supporting Information). CO gas and H_2_ gas were introduced in a controlled manner into the system from gas cylinders using calibrated mass flow controllers (MFC, Bronkhorst‐EL). The liquid and gaseous streams were combined in a simple four‐way inlet mixer (M) at room temperature. The mixer was connected to the tubular reactor through a fluoropolymer tubing (PFA, 1/8 in (1 in=2.54 cm) outer diameter (OD), 1/16 in internal diameter (ID)). The PFA tubing allowed visual inspection of the flow profile. The residence time (*t*_res_) reactor was a 60 mL stainless‐steel coil reactor (1/8 in OD, 1/16 in ID) heated on an aluminum heating block (Uniqsis FlowSyn). The reaction mixture exited the system through a short cooling loop and an adjustable back pressure regulator (BPR, Swagelok, 0–25 bar), which maintained a constant system pressure. A nitrogen purge was installed at the outlet. A pressure sensor (PS1) was integrated directly after one of the liquid pumps before entering the mixer to measure the system pressure.

Optimization experiments were performed with 4‐bromoanisole (**1 a)** as a model substrate under conditions close to those reported by Beller and co‐workers (Table 1) using Pd(OAc)_2_ and cata*CX*ium A^8^ but at residence times more appropriate to flow processing (<1 h). We expected that 4‐bromoanisole (**1 a**) would display relatively low reactivity towards the transformation due to the electron‐donating effect of the methoxy substituent because oxidative addition of the aryl bromide to the active palladium(0) species is typically the rate‐determining step in this transformation.^26^ For these reactions, 2.5 mmol of substrate and 0.75 equiv of base were dissolved in toluene and introduced as one feed, and Pd(OAc)_2_ (5 mol %) and cata*CX*ium A (15 mol %) were dissolved in toluene and introduced as the second feed, to provide homogeneous solutions. Pd^0^ precipitate formation occurred over time if the palladium catalyst and base were introduced in the same feed, from reduction of Pd^II^ to Pd^0^ particles.14b Sample loops and injection valves were used to load the liquid feeds. The liquid feeds were each pumped at equal flow rates. When the reaction was started, the injection valves were switched from the carrier solvent to the feeds for the reaction, and the feed mixtures were carried into the mixer, where they combined with CO and H_2_ to give a segmented gas‐liquid (Taylor) flow regime under the flow rates used in this study (Figure 2 b).

**Table 1:** Initial flow optimization of reductive carbonylation of 4‐bromoanisole (**1 a**).^[a]^ 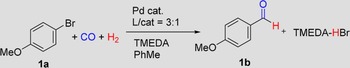

Entry	Total flow (liquid) [mL min^−1^]	CO [mL_n_ min^−1^]	H_2_ [mL_n_ min^−1^]	*T* [°C]	*P* [bar]	*t*_res_ [min]	Conv. **1 a** ^[b]^ [%]	Yield **1 b** ^[b]^ [%]	Selec. [%]
1	0.4	5	5	120	5	18	45	38	84
2	0.8	10	10	120	10	18	66	61	92
3	1.10	14	14	120	15	18	63	58	92
4	0.4	5	5	120	10	36	95	89	94
5	0.4	5	5	100	10	40	50	46	92
6	0.4	5	5	140	10	33	96	79	82
7^[c]^	0.4	5	5	120	10	36	1	0	0
8^[d]^	0.4	5	5	120	10	36	0	0	0

[a] Conditions: **1 a** (0.25 m) in anhydrous PhMe, 5 mol % Pd(OAc)_2_, 15 mol % cata*CX*ium A, 0.75 equiv. TMEDA, 15 mol % Ph_2_O as internal standard (IS). The liquid pumps were set at equal flow rates. Reactor coil was washed with 20 % aqueous nitric acid at 60 °C between experiments with the exception of entry 7. Conversion and yield determined by GC‐FID using Ph_2_O as IS, selectivity [%]=[product (mol]/1−starting material remaining [mol])×100 %. [b] Conversion and yield determined by GC‐FID using Ph_2_O as internal standard. [c] No Pd(OAc)_2_ within feed and no prereaction wash with aqueous nitric acid from previous run to remove deposited Pd black. [d] No Pd(OAc)_2_ added to feed.

Initially, the influence of temperature, pressure and gas flow rate were investigated to identify appropriate reaction conditions for the continuous‐flow reductive carbonylation of 4‐bromoanisole (**1 a**). For temperature and pressure optimization (Table 1), CO and H_2_ were fed in excess at equal flow rates to give ≈2.2 equiv of each gas relative to the substrate. The flow rates were adjusted at different pressures to provide comparative residence times. The conversion was relatively low at 5 bar pressure (entry 1), which was most likely caused by insufficient mass transfer of CO and H_2_ from the gas phase to the liquid phase. However, a drop‐in conversion was observed at 15 bar, probably due to catalyst deactivation by CO (entry 3). 10 bar pressure was identified to provide the best compromise between reaction rate and avoiding unwanted catalyst deactivation (entry 2). The reaction proceeded smoothly, giving 95 % conversion and 89 % desired product yield at 120 °C, 10 bar pressure, and 36 min residence time (entry 4). Conversion was significantly lower at 100 °C (entry 5) from a slower reaction rate. A higher reaction temperature resulted in higher conversion but did not improve yield due to accelerated catalyst decomposition (entry 6).

We knew from the outset that a well‐known phenomenon, and often unavoidable process, is the aggregation of Pd^0^ to form clusters that ultimately and irreversibly precipitate in the form of Pd black, which can then deposit onto the reactor wall.^27^ Deposited Pd can be recovered from a stainless‐steel coil by washing with 20 % aqueous nitric acid solution at 60 °C. The reactor coil was always washed between experiments (unless otherwise specified) with aqueous nitric acid solution to remove residual Pd. Washing with aqueous nitric acid solution demonstrated that considerable amounts of Pd were lost from solution and deposited onto the reactor wall. No Pd^0^ black particles were observed in the collected reaction mixtures, but Pd^0^ particles were observed when the solutions were kept overnight, indicating that not all Pd was deposited on the reactor channels. Running a reaction without fresh Pd(OAc)_2_ and without washing the deposited Pd indicated that the deposited Pd is catalytically inactive for the desired transformation (entry 7). The amount of deposited Pd was measured by inductively coupled plasma–mass spectrometry (ICP–MS) for the optimized conditions (vide infra). No desired transformation occurred in the absence of catalyst (entry 8).

We next investigated different catalytic systems that might provide better performance under the process‐intensified conditions utilized in a continuous‐flow environment (Table S1). Pd(OAc)_2_/cata*CX*ium A gave the highest conversion and yield compared to a selection of other phosphine ligands. The structure of the ligand appears to be very specific to the success of the reaction: two bulky alkyl groups and the long aliphatic tail are very important for the high efficiency of the catalytic system in terms of electron richness and steric shielding of the complexes.^28^ The catalytic system is critical to the reaction given the limited stability of the corresponding palladium(0) complexes in presence of base and CO, especially under high temperatures and pressures.

**Figure 3:**
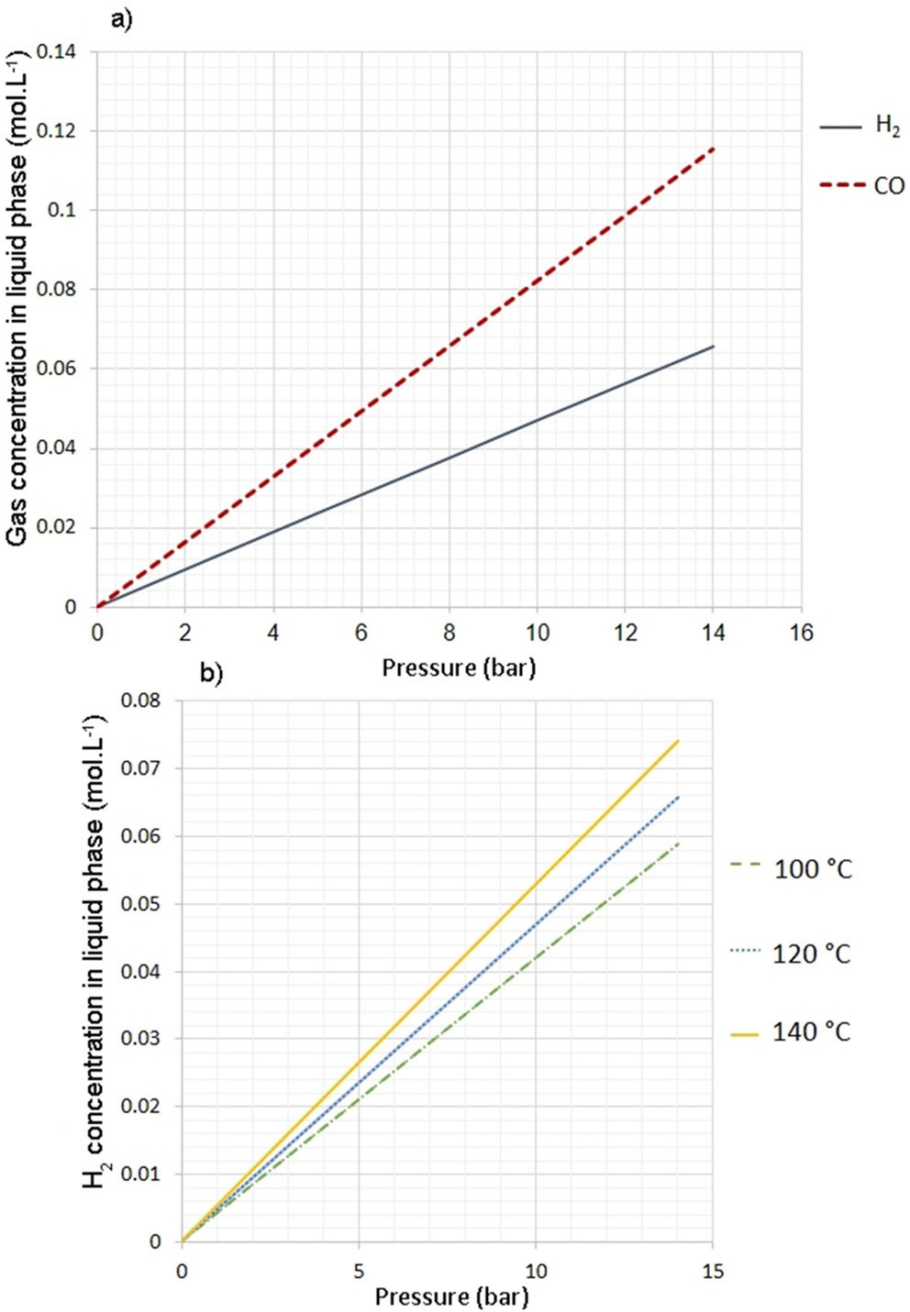
(a) Simulated concentration of H_2_ and CO dissolved in the liquid phase as a function of pressure at 120 °C; (b) Simulated concentration of H_2_ dissolved in the liquid phase at different temperatures and pressures.

The solubility of CO and H_2_ in toluene is relatively poor, 7.59×10^−3^ mol L^−1^ and 3.31×10^−3^ mol L^−1^, respectively, under standard conditions (20 °C and 1 bar). Thus, elevated pressures are necessary to dissolve a sufficient amount of the gases in solution to provide a reasonable reaction rate and therefore an appropriate residence time (<1 h) for processing within a tubular reactor. There are a number of reports measuring the solubility of CO and H_2_ in organic solvents; however data available for high temperature/pressure regimes are limited.^29^ Delmas and co‐workers measured the solubility of CO and H_2_ in toluene at relatively high temperatures (up to 100 °C) and pressures (up to 15 bar).29c The solubility of each gas in the liquid phase obeys Henry's law whereby the amount of dissolved gas is proportional to its partial pressure in the gas phase;^30^ therefore, the reaction rate should be directly proportional to the amount of gas dissolved in solution. The data from Delmas and co‐workers was simulated in DynoChem (Scaleup Systems) to predict the solubility of CO and H_2_ as a function of pressure. The solubility of CO in the liquid phase was approximately double that of H_2_ (Figure 3 a). H_2_ is competing with CO for dissolution in the liquid phase, and when these are used in equimolar amounts then the amount of H_2_ dissolved in solution will be lower than CO, so the effective concentration of CO will be higher. Increasing the reaction temperature would only have a small influence on the amount of dissolved H_2_ (Figure 3 b) whereas the solubility of CO in the liquid phase displayed only marginal temperature dependence (not shown).

The ability to carefully control the relative stoichiometric ratio of CO to H_2_ through varying the gas flow rates by using mass‐flow controllers is a key benefit of using continuous‐flow reactors (Table 2). The influence of gas stoichiometry has not previously been studied for reductive carbonylations. The conversion and yield significantly drops when CO and H_2_ were used at a 3:1 ratio, corresponding to 3.3 equiv of CO and 1.1 equiv of H_2_ (entries 1 and 2). Pd became easily deactivated by the CO because CO is a strong π‐acceptor ligand, forming palladium carbonyl clusters that can irreversibly form Pd black. The rate of oxidative addition is significantly reduced due to the loss of active catalyst. The clustering of Pd atoms is facile in the presence of CO resulting in nonactive palladium carbonyl complexes.^31^ One approach to prevent catalyst deactivation is to utilize CO at a close‐to‐stoichiometric amount.^32^ Very good conversion and yields were obtained at a 1:1 CO‐to‐H_2_ ratio (≈2.2 equiv of each gas). A CO‐to‐H_2_ ratio of 1:3 resulted in even better results with 99 % conversion and 98 % yield at 35 min residence time (entry 6). Under these conditions, the system becomes highly starved on CO towards the end of reaction. In these circumstances, at the beginning of the reactor there is a higher CO concentration while towards the end the concentration is very low because almost all CO has been consumed, thus improving process safety at the outlet due to the low concentration of CO.

**Table 2:** Optimization of gas stoichiometry for the reductive carbonylation of **1 a**.^[a]^

Entry	Total flow (liquid) [mL min^−1^]	CO/H_2_ ratio	CO [mL_n_ min^−1^]	H_2_ [mL_n_ min^−1^]	*t*_res_ [min]	Conv. **1 a** ^[b]^ [%]	Yield **1 b** ^[b]^ [%]	Selec. [%]
1	0.8	3:1	15	5	19	32	29	91
2	0.4	3:1	7.5	2.5	37	54	50	93
3	0.8	1:1	10	10	18	66	61	92
4	0.4	1:1	5.0	5.0	36	95	89	94
5	0.8	1:3	5.0	15	17	70	66	94
6	0.4	1:3	2.5	7.5	35	99	98	99

[a] Conditions: **1 a** (0.25 m) in anhydrous PhMe, 5 mol % Pd(OAc)_2_, 15 mol % cata*CX*ium A, 0.75 equiv TMEDA, 15 mol % Ph_2_O (IS), *T*=120 °C, *P*_sys_=10 bar, 30 min collection time. The liquid pumps were set at equal flow rates. Reactor coil was washed with 20 % aqueous nitric acid at 60 °C between experiments. [b] Conversion and yield determined by GC‐FID using Ph_2_O as IS.

The main limitation of cata*CX*ium A is that it is a proprietary ligand and therefore relatively expensive compared to many other phosphine ligands. Consequently, it was important to identify flow conditions that provided low ligand loadings to reduce costs and minimize waste. The catalyst and cata*CX*ium A loadings were lowered to more commercially viable levels, 1 and 3 mol %, respectively, for subsequent optimization. The conversion and yield dropped significantly on reducing the catalyst and ligand loadings, which could be improved by increasing the base to 3 equiv (Table S2, entry 6). Further optimization at a lower catalyst loading demonstrated that increasing the pressure from 10 to 12 bar resulted in a significant improvement in conversion and yield (Table 3, entries 3 and 5). However, there was a drop‐in conversion at 14 bar, indicating elevated catalyst deactivation from CO poisoning at higher pressures (entry 6). The optimal system pressure was identified as 12 bar (entry 7). The CO‐to‐H_2_ ratio became even more important when the catalyst and ligand loadings were lowered to more commercially viable levels, 1 and 3 mol % respectively (entries 1–3), compared to when higher catalyst and ligand loadings were used (Table 2). For the reaction in a segmented gas–liquid flow pattern, only a small excess of CO and H_2_ are needed whereas the reaction in a batch autoclave would require much more due to the reactor headspace.^8^ The low dosing of gases using continuous‐flow reactors is a key benefit of continuous‐flow reactors in terms of reducing usage and wastage and improving safety.

**Table 3:** Influence of CO/H_2_ ratio, pressure and residence time on conversion and yield.^*a*^

Entry	CO/H_2_ ratio	Total flow (liquid) [mL min^−1^]	CO [mL_n_ min^−1^]	H_2_ [mL_n_ min^−1^]	*P* [bar]	*t*_res_ [min]	Conv. **1 a** ^[b]^ [%]	Yield **1 b** ^[b]^ [%]	Selec. [%]
1	3:1	0.8	15	5	12	23	34	26	76
2	1:1	0.8	10	10	12	22	48	44	92
3	1:3	0.8	5	15	12	22	80	76	95
4	1:5	0.8	5	25	12	17	55	53	96
5	1:3	0.8	5	15	10	19	60	57	96
6	1:3	0.8	5	15	14	24	78	75	96
7	1:3	0.4	2.5	7.5	12	44	87	86	99

[a] Conditions: **1 a** (0.25 m) in anhydrous PhMe, 1 mol % Pd(OAc)_2_, 3 mol % cata*CX*ium A, 3 equiv TMEDA, 15 mol % Ph_2_O (IS), *T*=120 °C. Reactor coil was washed with 20 % aqueous nitric acid at 60 °C between experiments. [b] Conversion and yield determined by GC‐FID using Ph_2_O as internal standard.

Another strategy to prevent deactivation by CO is to utilize phosphine ligands at high ligand–Pd ratios (Table 4).^33^ A ligand‐to‐catalyst ratio of 2:1 resulted in a drop in conversion and yield (entry 1), probably due to catalyst poisoning from overcoordination of CO. The utilization of a ligand‐to‐catalyst ratio of 4:1 only resulted in a marginal improvement in yield (entry 4) compared to a 3:1 ratio. In contrast, an increase in the ligand‐to‐catalyst ratio to 5:1 retards the reaction and decreased the aldehyde yield to 71 % (entry 5). The slight increase in yield obtained when using a ligand‐to‐catalyst ratio of 4:1 compared to 3:1 often cannot be justified based on the increase in cost associated with using a higher ligand excess. We also investigated the use of stabilizing solvents to prevent the formation of black Pd^0^ particles. Even though environmentally their use should be minimized,^34^ polar aprotic solvents, for example, DMF and dimethylacetamide (DMA), can stabilize Pd^0^ species in solution.27b However, using these solvents as co‐solvents did not improve conversion or yield (Table S3) and therefore not investigated further.

**Table 4:** Optimization of catalyst and ligand loadings for reductive carbonylation.^[a]^

Entry	Cat. [mol %]	Ligand [mol %]	L/C ratio	*t*_res_ [min]	Conv. **1 a** ^[b]^ [%]	Yield **1 b** ^[b]^ [%]	Selec. [%]
1	1	2	2	44	72	70	97
2	1	3	3	45	90	86	96
3	1	4	4	46	91	89	98
4	1	5	5	46	74	71	96
5	0.5	2	4	47	58	53	91

[a] Conditions: **1 a** (0.25 m) in anhydrous PhMe, 3 equiv TMEDA, 15 mol % Ph_2_O (IS), *T*=120 °C, *P*_sys_=12 bar, 30 min collection time. Reactor coil was washed with 20 % aqueous nitric acid at 60 °C in‐between experiments. [b] Conversion and yield determined by GC‐FID using Ph_2_O as internal standard.

The applicability of the continuous‐flow protocol for the Pd‐catalyzed reductive carbonylation of aryl bromide substrates with syngas was demonstrated on a 3 mmol scale and is shown in Table 5 (18 examples). 4‐Bromobenzotrifluoride, possessing an electron‐deficient trifluoromethyl group, displayed high reactivity, giving full conversion and 97 % product yield (entry 2). The catalyst and ligand loadings could be lowered to 0.5 and 1.5 mol %, respectively, to afford the product in 78 % yield. 1‐Bromo‐4‐chlorobenzene also showed high reactivity giving quantitative conversion and 97 % product yield (entry 3). However, 1‐bromo‐4‐fluorobenzene (entry 5) displayed slightly lower reactivity than 1‐bromo‐4‐chlorobenzene. Aryl bromides bearing electron‐donating alkyl groups in the *para* position displayed moderate reactivity (entries 6 and 7). In particular, 4‐isobutylbenzaldehyde, an intermediate in the synthesis of ibuprofen, was afforded in 53 % yield (entry 7). 4‐Bromo‐*N*‐*N*‐dimethylaniline (entry 8), possessing an electron‐donating group, showed similar reactivity to 4‐bromoanisole. Heteroaryl bromides proved to be more challenging than bromobenzene‐substituted compounds (entries 9–12). Beller and co‐workers proposed that, in the case of 2‐bromopyridine, the catalyst was deactivated through the formation of inactive dimers after the oxidative addition step.^8^ We attempted to synthesize pyrimidine‐5‐carbaldehyde, a precursor for a myeloperoxidase (MPO) inhibitor,^35^ but only a poor 13 % yield was obtained (entry 10). In the instance of 3‐bromoquinoline, full conversion and 75 % yield were obtained, but dehalogenation of the starting material was also observed (entry 11). As the CO insertion step into the Pd−aryl bond can be slow as aromatic ring systems possess electron‐deficient substituents; this resulted in the competitive reduction of the aryl bromide in some cases (entries 11–18).^36^ A reduction in catalyst loading did not decrease the selectivity for the dehalogenated product. Aryl bromides containing carbonyl compounds displayed good reactivity under the flow protocol (entries 16–18). The conversions and yields were relatively stable for a 30 min operation time (Tables S2 and S3). Overall, the conversions and yields compared favorably to other reductive carbonylation batch protocols, see Table S6.

**Table 5:** Scope and limitations of reductive carbonylation flow protocol.^[a]^

Entry	Substrate (**a**)	Product (**b**)	Pd(OAc)_2_ [mol %]	cata*CX*ium A [mol %]	Conv.^[b]^ [%]	Yield^[b]^ [%]	Selec.^[b]^ [%]	Dehal. (**c**)^[b]^ [%]
1	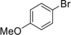	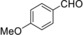	1	3	90	86	96	–
2	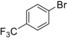	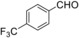	1	3	100	97	97	–
			0.5	1.5	81	78	96	–
3	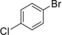	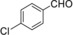	1	3	100	96	97	–
			0.5	1.5	58	56	97	–
4	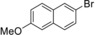	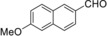	1	3	100	98	98	–
			0.5	1.5	99	95(84)	97	–
5	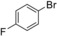	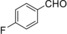	1	3	88	86	98	–
6	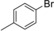	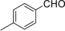	1	3	70	69(61)	99	–
7	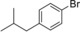	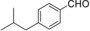	1	3	55	53	96	–
8	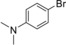	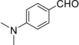	1	3	74	73(67)	99	–
9	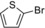	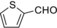	1	3	100	45	45	–
10	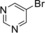	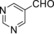	1	3	99	13	13	–
11	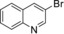	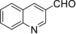	1	3	100	75	75	23
			0.5	1.5	92	71(66)	77	19
12	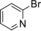	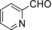	1	3	100	0	0	33
13	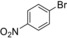	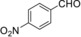	1	3	100	18	18	20
14	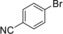	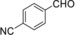	1	3	100	69(47)	69	28
15	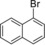	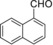	1	3	100	65(59)	65	34
16	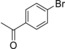	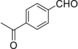	1	3	100	85(59)	85	12
17	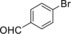	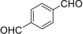	1	3	100	78(70)	78	20
18	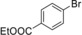	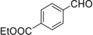	1	3	100	87(84)	87	12

[a] Reaction conditions: 3 mmol scale (hetero)arylbromide (0.25 m solution in anhydrous toluene), TMEDA (3 equiv), Ph_2_O (internal standard, 15 mol %), CO/H_2_=1:3, CO flow rate=2.5 mL_n_ min^−1^, H_2_ flow rate=7.5 mL_n_ min^−1^, catalyst feed flow rate=0.2 mL min^−1^, substrate feed flow rate=0.2 mL min^−1^, *P*_sys_=12 bar, *T*=120 °C, *t*_res_≈45 min. [b] Outlet was fractionated at 10 min intervals over a 30 min period, yields and conversion are average from 30 min collection time and determined by GC‐FID, see Tables S4 and S5 for conversions and yields for individual fractions. Molecular weights were confirmed by GC‐MS. Values given in parentheses are isolated yields after silica gel chromatography. The somewhat lower isolated yields compared to the GC yields in some cases may be due to the volatility of the product. Dehal.=dehalogenated product.

**Figure 4:**
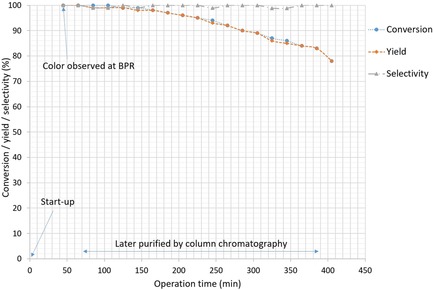
Long‐run profile. For reaction conditions and analytics, see Table 4, entry 4, with 0.5 mol % Pd(OAc)_2_ and 1.5 mol % cata*CX*ium A used for the long run. No samples were taken until color (reaction mixture) was observed at the BPR. Samples were fractionated at 20 min intervals. The first and last samples were less concentrated due to dilution by the “push‐out” solvent.

A scale‐up experiment was conducted for the reductive carbonylation of 2‐bromo‐6‐methoxynapthalene (**4 a**) to 6‐methoxy‐2‐naphthaldehyde (**4 b**), which is a possible intermediate in the synthesis of Naproxen, a nonsteroidal anti‐inflammatory drug.^37^ The total operation time was 415 min (from startup to shutdown), with the product being collected over 370 min (Figure 4). It was necessary to submerge the outlet tubing and BPR within an ultrasound bath and heat at 80 °C to prevent accumulation of a white solid near or at the BPR, which was from TMEDA–HBr salt precipitation. No pressure increase or fluctuation were observed for the duration of the experimental run. Only a very marginal drop in conversion or yield was observed for the first ≈170 min of runtime. However, the conversion and yield slowly decreased over the course of the run, with a drop of approximately 15 % over 350 min of runtime. The crude product was purified by column chromatography to give pure product in 85 % isolated yield, which enabled preparation of 3.8 g product, giving a throughput of 0.7 g h^−1^ from the continuous‐flow process.

Reactor contamination (fouling) is a critical issue in flow chemistry that is often overlooked.^38^ As discussed above, Pd^0^ species aggregate to generate Pd clusters, which ultimately irreversibly precipitate in the form of Pd black, which deposits on metal surfaces. ICP–MS analysis was conducted to measure the amount of Pd deposited on the reactor walls compared to the amount of Pd remaining in solution and to obtain a more thorough understanding of the slow decrease in conversion and yield over operation time for some substrates. ICP–MS analysis was conducted on samples fractionated at 20 min intervals for a separate experimental run for the reductive carbonylation of **4 a** over ≈2 h. Control experiments confirmed that the untreated steel material itself cannot catalyze the reductive carbonylation (vide supra). The amount of Pd measured in the collected reaction solution decreased over operation time, which indicated that the presence of existing “inactive” deposited catalyst accelerated the deposition of further catalyst (Table 6). ICP–MS measurements, along with the slow decrease in conversion and yield through the run, demonstrated that the rate of decomposition of catalyst increases over the duration of the run. The catalyst can either enter the catalytic cycle or aggregate to form initially soluble Pd clusters, which at some point will turn into insoluble Pd black. ICP–MS analysis confirmed that ≈80 % of the Pd was deposited on the walls over the duration of the experiment and that Pd is easily recovered by washing the reactor with 20 % aqueous nitric acid. The formation of Pd black is self‐catalyzed and leads to the withdrawal of Pd from the catalytic cycle. ICP–MS analysis also confirmed that no Fe or Co leached from the stainless steel into the reaction solution or from the aqueous nitric acid wash (Table S5).

**Table 6:** ICP–MS analysis of a flow experiment for the formylation of **4 a**.^[a]^

Entry	Sample [mg]	Pd determined [mg kg^−1^]	Pd determined [mg]	Pd expected [mg]	Pd determined [%]
fraction 1	253	2352	0.594	0.929	64
fraction 2	303	811	0.245	0.929	26
fraction 3	342	366	0.125	0.929	13
fraction 4	611	117	0.071	0.929	8
fraction 5	111	194	0.053	0.929	5
Aq. HNO_3_ wash	12 (mL)	290 (mg L^−1^)	3.48		
		**sum**	4.57	4.65	98

[a] For reaction conditions and analytics, see Table 4, entry 4, with 0.5 mol % Pd(OAc)_2_ and 1.5 mol % cata*CX*ium A. Samples were fractionated at 20 min intervals.

**Scheme 2:**
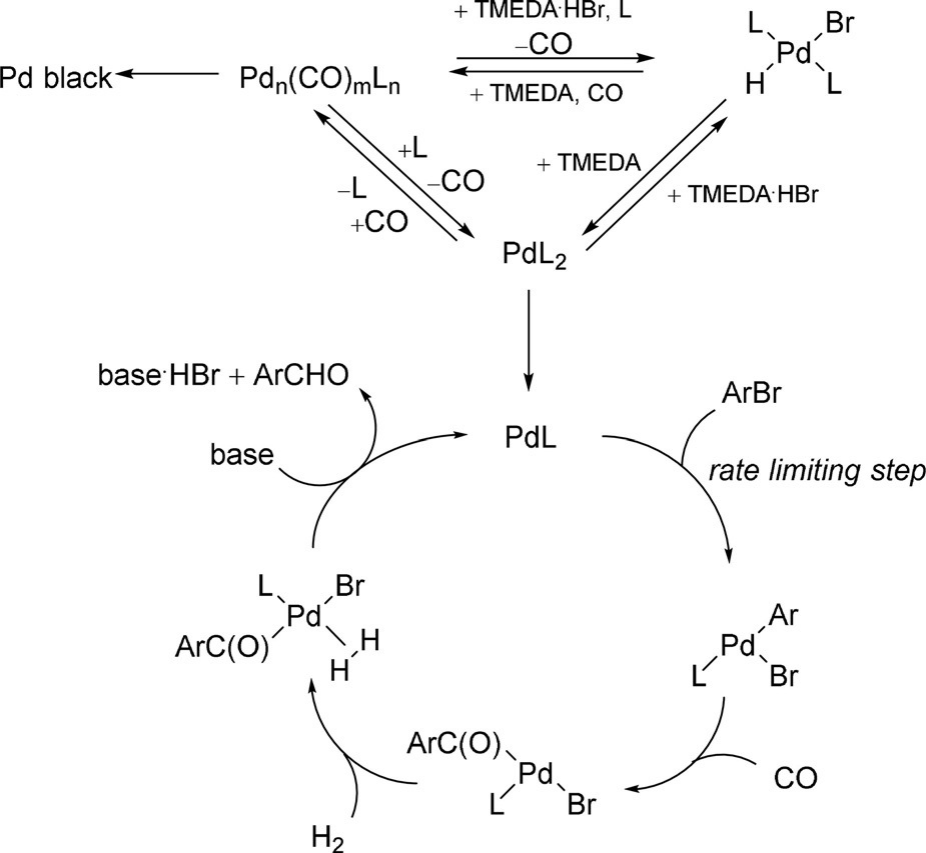
Mechanism for the Pd‐catalyzed reductive carbonylation of aryl bromides.

The catalytic cycle for Pd‐catalyzed formylation between aryl bromides and synthesis gas (CO/H_2_ 1:1) proposed by Beller and co‐workers is shown in Scheme 2.^26^ The catalytic cycle involves the oxidative addition of the aryl bromide with the active palladium(0) species, migratory insertion of CO into the Ar−Pd bond, coordination of a hydrogen molecule, and subsequent base‐mediated hydrogenolysis of the resulting acyl complex to give the desired aldehyde. The catalytic cycle is completed by the reaction of the palladium hydrobromide complex with base to regenerate Pd^0^. In the study by Beller and co‐workers, the carbonylpalladium(0) complex [Pd_*n*_(CO)_*m*_L_*n*_] and hydrobromide complex [Pd(Br)(H)L_2_] were identified as catalytic resting states; these complexes were not directly involved in the catalytic cycle. Consequently, the active catalyst [PdL] is always at low levels throughout the reaction, thus making the oxidative addition the rate‐determining step; therefore, aromatics containing an electron‐donating group are slower to react than the corresponding aromatics containing an electron‐withdrawing group. The efficiency of the Pd^0^ catalyst depends on the rate of the oxidative addition relative to the decomposition of Pd^0^; the agglomeration eventually leads to the formation of Pd black, which coats the reactor channels. The rate of the agglomeration process is second order in Pd or higher, whereas oxidative addition is usually first order in palladium(0), therefore the rate of Pd decomposition accelerates throughout operation time due to the presence of Pd on the reactor channels. ICP–MS showed that increasing amounts of catalyst were lost from solution over operation time, indicating that the presence of existing deposited Pd catalyzes the agglomeration process. The very slow decrease in conversion and yield observed over time for some substrates is caused by the increasing rate of catalyst decomposition over operation time.

## Conclusions

Green and sustainable chemical processes rely not only on effective chemistry but also on the implementation of reactor technologies that enhance reaction performance and overall safety. We have developed a continuous‐flow protocol for Pd‐catalyzed reductive carbonylation of (hetero)aryl bromides to aldehydes, with syngas as an inexpensive, atom‐economic, and environmentally friendly source of CO and H_2_. Relatively low catalyst loadings (0.5–1 mol %) and ligand loadings (1.5–3 mol %) provided moderate‐to‐excellent product yields. The reaction consumes only CO, H_2_, and base as stoichiometric reagents. The continuous‐flow protocol enabled the reaction time to be significantly reduced compared to the batch protocols available. For continuous‐flow reactions, gaseous reagents can be easily and accurately dosed into the system by using mass‐flow controllers, thus enabling precise control over the CO‐to‐H_2_ stoichiometric ratio. The investigation of gas stoichiometric ratio demonstrated that using CO/H_2_ at a 1:3 ratio prevented the formation of inactive Pd carbonyl clusters and therefore increased product yield. The flow reaction uses pure gases as feedstock to generate gas–liquid segmented flow patterns, which allows the reaction to be completed within 45 min residence time with much smaller excess (1.1 equiv of CO, 3.3 equiv of H_2_) of gases than required for batch processes. Under the flow conditions, at the end of the reactor the CO concentration is very low because almost all CO has been consumed, thus improving process safety at the outlet due to the low amount of CO present. The presence of deposited catalyst within the reactor was shown to have a negative effect on the reductive carbonylation. Inductively coupled plasma–mass spectrometry (ICP–MS) analysis demonstrated that the amount of deposited catalyst on the reactor channels increased over the duration of a run. The deposited catalyst could be recovered using an aqueous nitric acid wash. To improve safety, recent batch examples attempted to use liquid and solid reagents as gas surrogates for CO and H_2_. The continuous‐flow protocol with H_2_ and CO offers a safe, atom‐economic, and environmentally benign alternative to these gas‐surrogate procedures. The process developed herein is especially appealing for industrial applications, where atom economy, sustainability, reagent cost, and reagent availability and safety are important factors. Several key active pharmaceutical intermediates (APIs) were synthesized in a continuous and environmentally benign manner. In particular, a continuous‐flow protocol was operated for a 6 h run time to produce 3.8 g of an important active pharmaceutical intermediate. A major advantage of the continuous‐flow protocol is the ability to handle pure H_2_ and CO under process‐intensified conditions in a safe and scalable manner. Nevertheless, the long run and ICP–MS analysis demonstrated that there are challenges associated with catalyst decomposition over time. Further work is necessary to identify improved catalytic systems that allow the reactions to occur without any decomposition over time under process‐intensified conditions.

## Experimental Section

### General methods

NMR spectra were recorded on a 300 MHz instrument (75 MHz for ^13^C). Chemical shifts (*δ*) are expressed in ppm downfield from tetramethylsilane (TMS) as internal standard. The letters s, d, t, q, and m stand for singlet, doublet, triplet, quadruplet, and multiplet. Gas chromatography coupled with a flame ionization detector (GC–FID) analysis was performed using a HP5 column (30 m×0.250 mm×0.025 μm). After 1 min at 50 °C the temperature was increased stepped up to 80 °C at 2 °C min^−1^, then up to 300 °C at 25 °C min^−1^, and kept at 300 °C for 4 min. The detector gas for the flame ionization was H_2_ and compressed air (5.0 quality). GC–MS spectra were recorded using a HP5‐MS column (30 m×0.250 mm×0.25 μm) with helium as carrier gas (1 mL min^−1^ constant flow) coupled with a mass spectrometer (EI, 70 eV). After 1 min at 50 °C, the temperature was increased in 25 °C min^−1^ steps up to 300 °C and kept at 300 °C for 1 min. All solvents and chemicals were obtained from standard commercial vendors and were used without any further purification. All compounds synthesized herein are known in the literature.

CAUTION: CO is highly toxic and flammable, therefore extreme care must be taken when handling. H_2_ is extremely flammable. CO alarms must be installed and N_2_ purge used at the outlet. All equipment must be set up in a well‐ventilated fume hood. A thorough safety assessment should be made before conducting any experiments.

### Representative procedure for reductive carbonylation of (hetero)aryl bromides

Data are reported in Table 5. Flow experiments were performed using the continuous‐flow setup depicted in Figure 2 (also see Figure S1 for a labeled image). The continuous‐flow setup is described in detail in the Results and Discussion section. The solution of substrate (0.5 m in PhMe, corresponding to 0.25 m within the reactor), tetramethylethylenediamine (TMEDA) (3 equiv), and diphenylether (15 mol %) as an internal standard (stream 1) and Pd(OAc)_2_ (1 mol % or 0.5 mol %) and cata*CX*ium A (3 mol % or 1.5 mol %) in PhMe (stream 2) were loaded into their corresponding sample loops. The liquid feeds were pumped using two high‐pressure liquid pumps (HPLC) (P, Uniqsis) with a flow rate of 0.2 mL min^−1^ for each pump, using toluene as a carrier solvent. The flow rates of the gas streams were measured and controlled by two calibrated mass‐flow controllers (MFCs) using flow rates of 2.5 (CO) and 7.5 mL_n_ min^−1^ (gas flow rates were measured in units of mL_n_ min^−1^, where n represents measurement under standard conditions, i.e., *T*_n_=0 °C, *P*_n_=1.01 bar) (H_2_). The system was maintained at 120 °C and 12 bar pressure to provide ≈45 min residence time. The residence time was measured from the four streams mixing at the mixer until color was observed at the BPR. The liquid pump flow rates, temperature, and pressure were measured and monitored by the control platform of the pumping system. Once color was observed at the BPR, fractions were collected for 10 min intervals over a 40 min period. Collection was stopped once no color was observed at the BPR. Yields and conversion were determined by GC‐FID using diphenylether as internal standard and the reported values are an average from 30 min collection time. In some cases fractions were combined for purification by silica gel chromatography.

**4‐Methylbenzaldehyde (6 b)**: The title compound was prepared according to the general procedure as a colorless oil in 61 % yield after silica gel chromatography (petroleum ether/EtOAc=100:0 to 99:1 then isocratic petroleum ether/EtOAc=99:1). ^1^H NMR (300 MHz, CDCl_3_): *δ*=9.96 (s, 1 H), 7.83–7.72 (m, 2 H), 7.33 (d, *J* 8.0 Hz, 2 H), 2.44 ppm (s, 3 H); ^13^C NMR (75 MHz, CDCl_3_): *δ*=192.1, 145.7, 134.3, 130.0, 129.8, 22.0 (Ref. 39).

**4‐Dimethylaminobenzaldehyde (8 b)**: The title compound was prepared according to the general procedure as white crystals in 67 % yield after silica gel chromatography (petroleum ether/EtOAc=100:0 to 85:15 then isocratic petroleum ether/EtOAc=85:15). m.p. 74.2–74.6 °C (Ref. 41 72–73 °C); ^1^H NMR (300 MHz, CDCl_3_): *δ*=9.74 (s, 1 H), 7.78–7.68 (m, 2 H), 6.76–6.65 (m, 2 H), 3.08 ppm (s, 6 H). ^13^C NMR (75 MHz, CDCl_3_): *δ*=190.5, 154.4, 132.1, 125.3, 111.1, 40.2 ppm (Ref. 39).

**Quinoline‐3‐carbaldehyde (11 b)**: The title compound was prepared according to the general procedure as an off‐white solid in 66 % yield after silica gel chromatography (petroleum ether/EtOAc=100:0 to 80:20 then isocratic petroleum ether/EtOAc=80:20). m.p. 69.6–69.9 °C (Ref. 39 70 °C); ^1^H NMR (300 MHz, CDCl_3_): *δ*=10.26 (s, 1 H), 9.37 (d, *J* 1.8 Hz, 1 H), 8.64 (d, *J* 1.8 Hz, 1 H), 8.20 (d, *J* 8.5 Hz, 1 H), 8.00 (d, *J* 8.1 Hz, 1 H), 7.89 (ddd, *J* 8.5, 7.0, 1.5 Hz, 1 H), 7.67 ppm (ddd, *J* 8.1, 7.0, 1.5 Hz, 1 H); ^13^C NMR (75 MHz, CDCl_3_): *δ*=190.9, 150.7, 149.3, 140.3, 132.8, 129.8, 129.6, 128.7, 128.0, 127.2 ppm (Ref. 39).

**4‐Cyanobenzaldehyde (14 b)**: The title compound was prepared according to the general procedure as white crystals in 47 % yield after silica gel chromatography (petroleum ether/EtOAc=100:0 to 92:8 then isocratic petroleum ether/EtOAc=92:8). m.p. 100.8–101.3 °C (Ref. 42 100–101 °C); ^1^H NMR (300 MHz, CDCl_3_): *δ*=10.09 (s, 1 H), 8.05–7.94 (m, 2 H), 7.89–7.80 ppm (m, 2 H); ^13^C NMR (75 MHz, CDCl_3_): *δ*=190.8, 138.8, 133.0, 130.0, 117.9, 117.7 ppm (Ref. 39).

**1‐Naphthaldehyde (15 b)**: The title compound was prepared according to the general procedure as a yellow liquid in 59 % yield after silica gel chromatography (petroleum ether/EtOAc=100:0 to 99:1 then isocratic petroleum ether/EtOAc=99:1). ^1^H NMR (300 MHz, CDCl_3_): *δ*=10.41 (s, 1 H), 9.26 (d, *J* 8.8 Hz, 1 H), 8.11 (d, *J* 8.2 Hz, 1 H), 8.00 (dd, *J* 7.0, 1.3 Hz, 1 H), 7.93 (d, *J* 8.2 Hz, 1 H), 7.70 (ddd, *J* 8.5, 7.0, 1.5 Hz, 1 H), 7.67–7.62 (m, 1 H), 7.62–7.56 ppm (m, 1 H); ^13^C NMR (75 MHz, CDCl_3_): *δ*=193.7, 136.8, 135.4, 133.9, 131.5, 130.7, 129.2, 128.6, 127.1, 125.1, 125.0 ppm (Ref. 39).

**4‐Acetylbenzaldehyde (16 b)**: The title compound was prepared according to the general procedure as a pale‐yellow low melting solid in 59 % yield after silica gel chromatography (petroleum ether/EtOAc=100:0 to 91:9 then isocratic petroleum ether/EtOAc=91:9). ^1^H NMR (300 MHz, CDCl_3_): *δ*=10.10 (s, 1 H), 8.14–8.05 (m, 2 H), 8.01–7.94 (m, 2 H), 2.66 ppm (s, 3 H); ^13^C NMR (75 MHz, CDCl_3_): *δ*=197.5, 191.7, 141.3, 139.2, 130.0, 128.9, 27.1 ppm (Ref. 40).

**Benzene‐1,4‐dicarboxaldehyde (17 b)**: The title compound was prepared according to the general procedure as white crystals in 70 % yield after silica gel chromatography (petroleum ether/EtOAc=100:0 to 90:10 then isocratic petroleum ether/EtOAc=90:10). m.p. 116.8–117.3 °C (Ref. 39 115 °C); ^1^H NMR (300 MHz, CDCl_3_): *δ*=10.13 (s, 2 H), 8.05 ppm (s, 4 H); ^13^C NMR (75 MHz, CDCl_3_): *δ*=191.7, 140.1, 130.3 ppm (Ref. 39).

**4‐Ethoxycarbonylbenzaldehyde (18 b)**. The title compound was prepared according to the general procedure as a colorless oil in 84 % yield after silica gel chromatography (petroleum ether/EtOAc=100:0 to 95:5 then isocratic petroleum ether/EtOAc=95:5). ^1^H NMR (300 MHz, CDCl_3_): *δ*=10.09 (s, 1 H), 8.23–8.14 (m. 2 H), 7.98–7.88 (m, 2 H), 4.40 (q, *J* 7.1 Hz, 2 H), 1.40 ppm (t, *J* 7.1 Hz, 3 H). ^13^C NMR (75 MHz, CDCl_3_): *δ*=191.8, 165.7, 139.2, 135.5, 130.2, 129.6, 61.7, 14.4 ppm (Ref. 39).

### Long run procedure for the preparation of 6‐methoxy‐2‐naphthaldehyde (4 b) using continuous‐flow

**Preparation of catalyst feed**: Pd(OAc)_2_ (42.1 mg, 0.188 mmol, 0.5 mol %) and cata*CX*ium A (201.7 mg, 0.563 mmol, 1.5 mol %) were weighed into 100 mL two‐necked round‐bottom flask containing a magnetic stirrer. The flask was enclosed with a rubber septum, and a balloon filled with argon was attached. Anhydrous toluene (75 mL) was added to the flask, and the mixture was stirred for 15 min to provide a homogeneous yellow solution (75 mL).

**Preparation of substrate feed**: **4 a** (7.113 g, 30 mmol, 1 equiv) was weighed into a 100 mL two‐necked round‐bottom flask containing a magnetic stirrer. The flask was enclosed with a rubber septum, and a balloon filled with argon was attached. Subsequently, Ph_2_O (709 μL, 4.5 mmol, 15 mol %), TMEDA (13.5 mL, 90 mmol, 3 equiv), and anhydrous toluene (60 mL) were added under argon atmosphere. The resulting mixture was stirred for 15 min to provide a homogeneous pale‐yellow solution (80.5 mL).

**Flow procedure**: Flow experiments were performed using the continuous‐flow setup depicted in Figure 1 (also see Figure S1 for a labeled image). Before the reaction, the entire flow system was washed with 20 % aqueous nitric acid solution at 60 °C to ensure that no residual Pd was still deposited on the reactor channels and then subsequently washed with acetonitrile and then toluene. Calibrated MFCs (EL‐Bronkhorst) were set to the desired flow rates (2.5 mL_n_ min^−1^ for CO and 7.5 mL_n_ min^−1^ for H_2_), and gases started to flow into the reactor. The pressure was slowly increased at the BPR (Swagelok). When the system reached 3 bar, the liquid pumps were started, each liquid pump was operated at 0.2 mL min^−1^, corresponding to a total liquid flow rate of 0.4 mL min^−1^, with both pumping toluene. The pressure was slowly increased to 12 bar, and the temperature set to 120 °C. Once at the desired temperature and pressure, the streams were switched to the feed solutions. The feeds were introduced directly through the pumps. The streams were mixed using a four‐way inlet mixer at room temperature to give a segmented flow regime and then flowed through the reactor. The residence time ≈45 min was the time measured from the four streams mixing at the mixer until color was observed at the BPR. The tubing after the stainless‐steel coil and the Swagelok BPR were immersed in an ultrasound bath and heated at 80 °C for the duration of the experiment to prevent accumulation of solids in front of and within the BPR. The feed solutions were pumped for 350 min, then toluene was pumped for the remaining time as a carrier solvent. A total of 18 fractions were collected, a fraction was collected every 20 min (approx. 8 mL), and conversion and yield measured by GC‐FID using diphenylether as an internal standard (see Figure 4 for conversion and yield over operation time).

**Isolation procedure**: All fractions were combined (except the first and last) to give 128 mL (max. yield would give 23.9 mmol based on 64 mL substrate feed), and the volatiles were removed under reduced pressure. The residue was dissolved in EtOAc and absorbed on silica gel (43 to 60 μm particle size). Purification of the crude product by silica chromatography (petroleum ether/EtOAc=100:0 to 95:5 then isocratic petroleum ether/EtOAc=95:5). Subsequent removal of solvent under reduced pressure afforded **4 b** (85 % yield, 3.77 g, 20.2 mmol) as white crystals. m.p. 83.4–83.8 °C (Ref. 43 80–82 °C); ^1^H NMR (300 MHz, CDCl_3_): *δ*=10.01 (s, 1 H), 8.17 (d, *J* 1.7 Hz, 1 H), 7.84 (dd, *J* 8.5, 1.7 Hz, 1 H), 7.81 (d, *J* 9.0 Hz, 1 H), 7.73 (d, *J* 8.5 Hz, 1 H), 7.16 (dd, *J* 9.0, 2.5 Hz, 1 H,), 7.10 (d, *J* 2.5 Hz, 1 H), 3.88 ppm (s, 3 H); ^13^C NMR (75 MHz, CDCl_3_): *δ*=192.2, 160.4, 138.4, 134.4, 132.5, 131.2, 128.1, 127.9, 123.8, 120.1, 106.2, 55.6 ppm (Ref. 39).

**ICP–MS**: The amount of Pd deposited within the reactor compared to remaining in solution was determined by ICP–MS analysis. The crude reaction solution collected from the reactor was evaporated under reduced pressure to remove all volatile compounds. The resulting residue was dissolved in acetonitrile/concentrated nitric acid to give a homogeneous solution. The deposited Pd from the reactor channels was collected by washing with 20 % aqueous nitric acid at 60 °C. The solutions were diluted with nitric acid to 40 mL and placed in a vial for microwave digestion. Microwave‐assisted acid digestion was carried out in an MLS UltraClave IV instrument. The temperature was ramped up in 30 min to 250 °C and kept at this temperature for a further 30 min. After appropriate dilution Pd was quantitatively determined at *m*/*z* 105 with an Agilent 7500ce inductively coupled plasma–mass spectrometer. A calibration was performed with an external calibration curve established from 1000 g_Pd_ L^−1^ standard (CPI International). Indium served as the internal standard.

## Conflict of interest

*The authors declare no conflict of interest*.

## Supporting information

As a service to our authors and readers, this journal provides supporting information supplied by the authors. Such materials are peer reviewed and may be re‐organized for online delivery, but are not copy‐edited or typeset. Technical support issues arising from supporting information (other than missing files) should be addressed to the authors.

Supplementary
