# Supplementary material for: Continuous‐flow Synthesis of Aryl Aldehydes by Pd‐catalyzed Formylation of Aryl Bromides Using Carbon Monoxide and Hydrogen
Source: ChemSusChem. 2018 Nov 13;12(1):326–37. doi: 10.1002/cssc.201802261 (PMC6582436; doi:10.1002/cssc.201802261)
Supplement: Supplementary file 1 — Supplementary [file CSSC-12-326-s001.pdf]

CHEMISTRY & SUSTAINABILITY
CHEMSUSCHEM
ENERGY & MATERIALS

Supporting Information

Continuous-flow Synthesis of Aryl Aldehydes by Pd-catalyzed Formylation of Aryl Bromides Using Carbon Monoxide and Hydrogen

Christopher A. Hone,[a, b] Pavol Lopatka,[b] Rachel Munday,[c] Anne O'Kearney-McMullan,[c] and C. Oliver Kappe*[a, b]

cssc_201802261_sm_miscellaneous_information.pdf

Supporting Information

Contents

Figure S1. Labelled image of the flow reactor setup. 2

Table S1. Conversions and yields for the different fractions – part (i). 4

Table S2. Conversions and yields for the different fractions – part (ii). 5

Table S3. Comparison of the flow protocol to previously reported batch protocols. 6

Table S4. ICPMS analysis for residual metals 7

1H-NMR and 13C-NMR Spectra 7

Figure S1. Labelled image of the flow reactor setup.

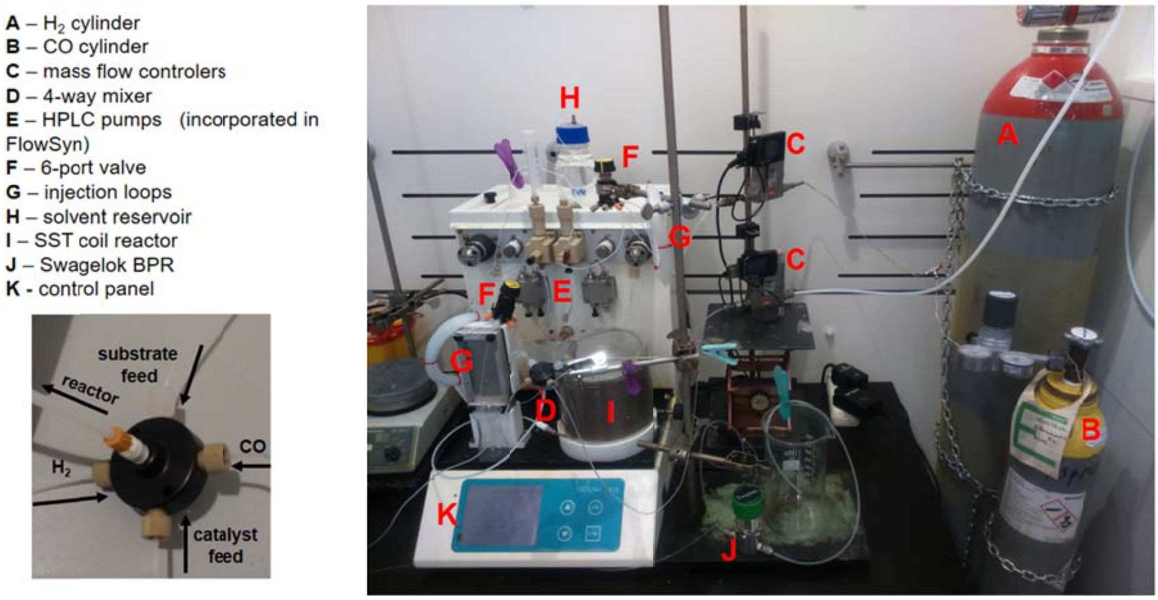

Table S1. Investigation of different catalyst/ligand systems.[a]

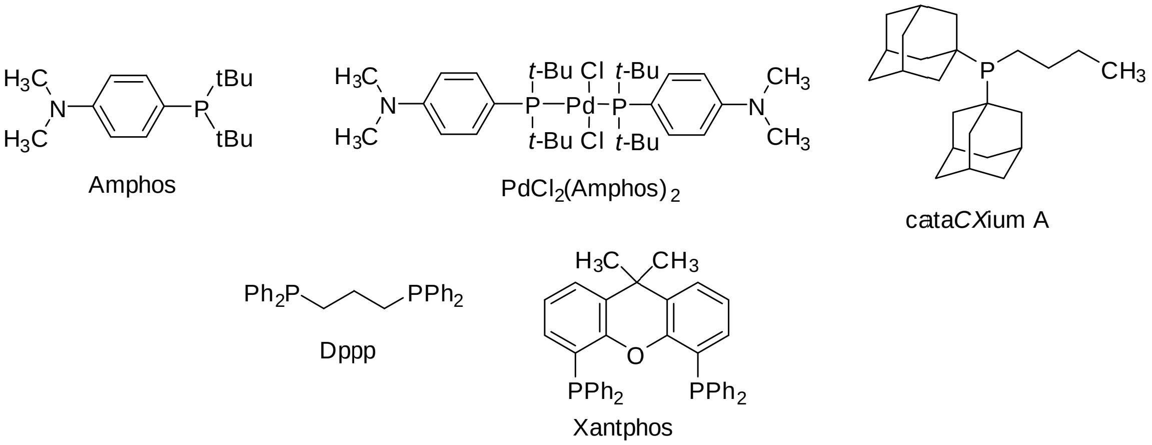

Entry	Catalyst	Ligand	Conv. **1a** [%][b]	Yield **1b** [%][b]	Selec. [%]
1	–	–	1	0	0
2	$Pd(OAc)_2$	cata*CX*ium A	95	89	94
4	$Pd(OAc)_2$	Amphos	70	41	59
5	$Pd(PPh_3)_4$	–	25	5	20
6	$Pd(OAc)_2$	Xantphos	50	10	20
7	Pd(OAc)2	Dppp	10	5	50
8[c]	$PdCl_2(Amphos)_2$	–	17	0	0

[a] Conditions: **1a** (0.25 M) in anhydrous PhMe, 5 mol% Pd cat., 15 mol% ligand, 0.75 equiv. TMEDA, 15 mol% Ph_2O (IS), T = 120 °C, P_{sys} = 10 bar, t_{res} ~36 min, 30 min collection time, catalyst feed flow rate = substrate feed flow rate = 0.2 mL/min, H_2 flow rate = CO flow rate = 5 mL_n/min. Conversion and yield determined by GC-FID using Ph_2O (IS). [c] $PdCl_2(Amphos)_2$ in toluene formed a milky suspension therefore was filtered before injection.

Table S2. Base equivalent optimization at lower catalyst and ligand loadings.[a]

Entry	Cat. [mol%]	Ligand [mol%]	P [bar]	TMEDA [equiv]	t_{res} [min]	Conv. **1a** [%][b]	Yield **1b** [%][b]	Selec. [%]
1	5	15	5	0.75	18	45	38	84
2	1	3	5	0.75	22	12	5	72
3	5	15	10	0.75	36	95	89	94
4	1	3	10	0.75	36	34	32	94
5	1	3	10	1.50	36	42	39	93
6	1	3	10	3.00	36	50	46	92
7	1	3	10	4.50	36	51	46	90

[a] Conditions: **1a** (0.25 M) in anhydrous PhMe, 15 mol% Ph_2O (IS), T = 120 °C, 30 min collection time, entries 1 and 2: catalyst feed flow rate = substrate feed flow rate = 0.4 mL/min, H_2 flow rate = CO flow rate = 10 mL_n/min. entries 3 to 6: catalyst feed flow rate = substrate feed flow rate = 0.2 mL/min, H_2 flow rate = CO flow rate = 5 mL_n/min. Reactor coil was washed with 20% aqueous nitric acid at 60 °C in-between experiments. Conversion and yield determined by GC-FID using Ph_2O (IS).

Table S3. Trial of co-solvents.

Entry	Solvent	t_{res} [min]	Conv. **1** [%][b]	Yield **2** [%][b]	Selec. [%]
1	PhMe	46	90	86	96
2	PhMe/DMF (9:1)	46	82	80	98
3	PhMe/DMA (9:1)	46	73	72	99

3 mmol scale (hetero)arylbromide (0.25 M solution in anhydrous toluene), TMEDA (3 equiv.), Ph_2O (internal standard, 15 mol%), CO:H_2 = 1:3, CO flow rate = 2.5 mL_n/min, H_2 flow rate = 7.5 mL_n/min, catalyst feed flow rate = 0.2 mL/min, substrate feed flow rate = 0.2 mL/min, P_{sys} = 12 bar, T = 120 °C, t_{res} ~45 min.

Table S4. Conversions and yields for the different fractions – part (i).[a]

Entry	Product	Cat/Lig. [mol %]	Fraction 1		Fraction 2		Fraction 3		Overall		Isolated yield [%]
			Conv. [%]	Yield [%]	Conv. [%]	Yield [%]	Conv. [%]	Yield [%]	Conv. [%]	Yield [%]	
1	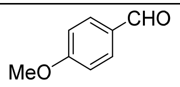	1/4	98	95	93	91	82	81	91	89	-
		1/3	95	88	92	89	82	81	90	86	-
2	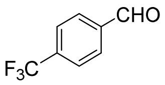	1/3	99	98	100	98	100	95	100	97	5
		0.5/1.5	78	75	82	80	84	80	81	78	-
3	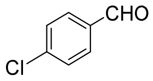	1/3	99	95	99	96	98	97	99	96	-
		0.5/1.5	54	53	61	59	59	55	58	56	-
4	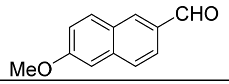	1/3	100	99	100	96	100	99	100	98	-
		0.5/1.5	99	96	100	96	99	94	99	95	84
5	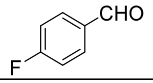	1/3	94	93	89	88	80	78	88	86	45
6	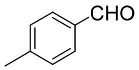	1/3	75	74	74	74	62	60	70	69	61
7	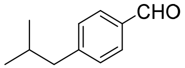	1/3	56	55	60	59	50	44	55	53	-
8	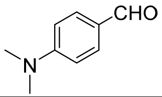	1/3	84	82	75	74	64	62	74	73	67
9	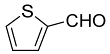	1/3	100	44	100	45	100	46	100	45	21
10	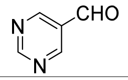	1/3	98	13	99	13	100	14	99	13	-

[a] Reaction conditions: 3 mmol scale (hetero)arylbromide (0.25 M solution in anhydrous toluene), TMEDA (3 equiv.), Ph_2O (internal standard, 15 mol%), CO:H_2 = 1:3, CO flow rate = 2.5 mL_n/min, H_2 flow rate = 7.5 mL_n/min, catalyst feed flow rate = 0.2 mL/min, substrate feed flow rate = 0.2 mL/min, P_{sys} = 12 bar, T = 120 °C, t_{res} ~45 min. [b] outlet was fractionated at 10 min intervals over a 30 min period, yields and conversion are determined by GC-FID,Molecular weights were confirmed by GC-MS. Isolated yields after silica gel chromatography.

Table S5. Conversions and yields for the different fractions – part (ii).[a]

Entry	Product	By-product	Cat/Lig. [mol %]	Fraction 1			Fraction 2			Fraction 3			Overall			Isolated yield	
				Conv [%]	Yield P [%]	Yield BP [%]	Conv [%]	Yield P [%]	Yield BP [%]	Conv [%]	Yield P [%]	Yield BP [%]	Conv [%]	Yield P [%]	Yield BP [%]	P [%]	BP [%]
11	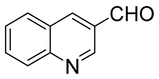	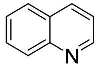	1/3	99	74	22	100	76	23	100	76	23	100	75	23	66	17
			0.5/1.5	89	69	18	93	72	19	95	71	19	92	71	19	-	-
12	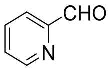	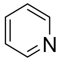	1/3	100	0	32	100	0	35	100	0	33	100	0	33	-	-
13	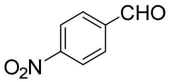	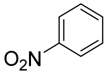	1/3	100	18	20	100	17	20	100	18	21	100	18	20	-	-
14	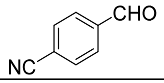	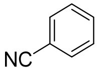	1/3	100	69	28	100	70	27	100	69	28	100	69	28	47	0
15	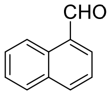	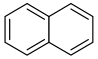	1/3	100	65	34	100	65	34	100	65	34	100	65	34		
16	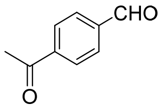	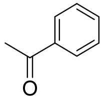	1/3	100	86	12	100	83	12	100	86	11	100	85	12	59	3
17	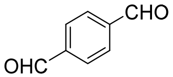	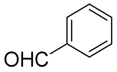	1/3	100	79	20	100	77	21	100	78	20	100	78	20	70	7
18	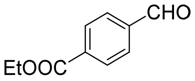	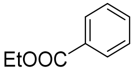	1/3	100	87	12	100	87	12	100	87	13	100	87	12	84	10

[a] Reaction conditions: 3 mmol scale (hetero)arylbromide (0.25 M solution in anhydrous toluene), TMEDA (3 equiv.), Ph_2O (internal standard, 15 mol%), CO:H_2 = 1:3, CO flow rate = 2.5 mL_n/min, H_2 flow rate = 7.5 mL_n/min, catalyst feed flow rate = 0.2 mL/min, substrate feed flow rate = 0.2 mL/min, P_{sys} = 12 bar, T = 120 °C, t_{res} ~45 min. [b] outlet was fractionated at 10 min intervals over a 30 min period, yields and conversion are determined by GC-FID,Molecular weights were confirmed by GC-MS. Isolated yields after silica gel chromatography.

Table S6. Comparison of the flow protocol to previously reported batch protocols.[a]

Entry	Product (**b**)	Conversion/Yield/Isolated Yield [%][b]							
		this work	1	2	3	4	5	6	7
1	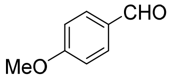	91/89/-	100/96/-	-/-/72	-/93/87	99/96/-	-/-/89	-/-/72	81/81/-
2	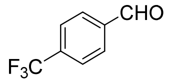	100/97/5	100/84/-	-/-/60	-	100/65/-	-/-/61	-/-/68	100/78/-
3	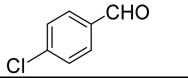	99/96/-	100/89/-	-/-/66	-	-	-/-/75	-/-/91	-
4	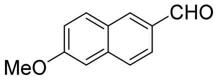	99/95/84	100/99/-	-/-/61	-	99/93/-	-	-	-
5	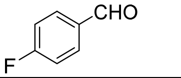	88/86/45	98/89/-	-/-/65	-	99/88/-	-/-/78	-/-/76	-
6	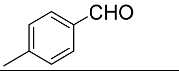	70/69/61	-	-/-/75	-/93/78	100/90/-	-/-/81	-/-/76	92/92/-
7	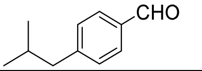	55/53/-	-	-	-	-	-	-	-
8	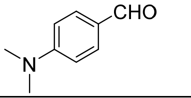	74/73/67	99/98/-	-/-/47	-/-/82	99/95/-	-	-/-/90	-
9	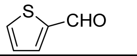	100/45/21	99/66/-	-/-/47	-	-	-/-/73	-	-
10	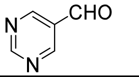	99/13/-	-	-	-	-	-	-	-
11	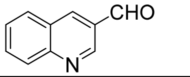	100/75/66	-	-	-/-/70	-	-	-/-/62	-
12	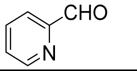	100/0/-	21/7/-	-	-/49/45	-	-	-/-/53	100/100/-
13	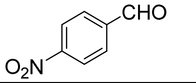	100/18/-	8/0/-	-	-	-	-	-	100/4/-
14	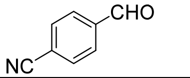	100/69/47	99/74/-	-	-/-/60	90/77/-	-	-/-/77	100/79/-
15	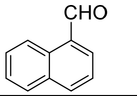	100/65/59	100/86/-	-/-/54	-/-/66	91/83/-	-/-/76	-	-
16	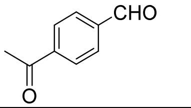	100/85/59	100/88/-	-	-	-	-	-/-/73	100/82/-
17	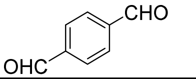	100/78/70	-	-/-/60	-/-/65	84/68/-	-/-/65	-	-
18	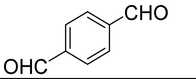	100/87/84	-	-	-/-/72	-	-	-	-

[a] Reaction conditions are as for Table 7. The results shown in red are "this work" described in this manuscript. [b] Conversion and yield comparisons and the protocols are provided in the references given below.

1. A General and Efficient Method for the Formylation of Aryl and Heteroaryl Bromides (CO/H_2). S. Klaus, H. Neumann, A. Zapf, D. Strübing, S. Hübner, J. Almena, T. Riermeier, P. Gross, M. Sarich, W.-R. Krahnert, K. Rossen, M. Beller, *Angew. Chem. Int. Ed.* **2006**, *45*, 154–158.

2. Palladium-Catalyzed Carbonylations of Aryl Bromides using Paraformaldehyde: Synthesis of Aldehydes and Esters ($(CH_2O)_n/Et_3SiH$). K. Natte, A. Dumrath, H. Neumann, M. Beller, *Angew. Chem. Int. Ed.* **2014**, *53*, 10090–10094.

3. Palladium-Catalyzed Reductive Carbonylation of Aryl Halides with N-Formylsaccharin as a CO Source (*N*-formylsaccharin/Et_3SiH) T. Uead, H. Konishi, K. Manabe, *Angew. Chem. Int. Ed.* **2013**, *52*, 8611–8615.

4. Palladium-Catalyzed Reductive Carbonylation of Aryl Bromides with Phosphinite Ligands (CO/H_2). H. Neumann, R. Kadyrov, X.-F. Wu M. Beller, *Chem. Asian J.* **2012**, *7*, 2213–2216.

5. An Efficient and General Method for Formylation of Aryl Bromides with CO_2 and Poly(methylhydrosiloxane) (CO_2/ PMHS). B. Yu, Z. Yang, Y. Zhao, L. Hao, H. Zhang, X. Gao, B. Han, Z. Liu, *Chem. Eur. J.* **2016**, *22*, 1097–1102.

6. Convenient Palladium□Catalyzed Reductive Carbonylation of Aryl Bromides Under Gas□ Free Conditions (HCOOH/HCOONa). J. Ying, L.-Y. Fu, C. Zhou, X. Qi, J.-B. Peng, X-.F. Wu, *Eur. J. Org. Chem.* **2018**, 2780–2783.

7. Reductive Carbonylation − an Efficient and Practical Catalytic Route for the Conversion of Aryl Halides to Aldehydes (CO/Et_3SiH). L. Ashfield, C. F. J. Barnard, *Org. Process Res. Dev.* **2007**, *11*, 39–43.

Table S7. ICPMS analysis for residual metals for before, during and after reaction.

Entry	Cr [mg/mL]	Mn [mg/mL]	Fe [mg/mL]	Co [mg/mL]	Ni [mg/mL]
catalyst feed 1	1.11	0.40	255	0.51	1.81
catalyst feed 2	0.69	0.22	223	0.46	1.30
catalyst feed 3	0.55	0.14	190	0.38	1.03
Fraction 1	0.80	0.16	28	0.13	11.34
Fraction 2	0.14	0.04	7	0.020	0.58
Fraction 3	0.73	0.22	30	0.074	1.80
Fraction 4	0.41	0.12	20	0.045	0.87
Fraction 5	0.32	0.15	24	0.030	1.10
aq. HNO3 wash	7.11	0.50	29	0.095	5.07
aq. HNO3 wash	0.59	0.08	1.7	0.005	0.25

1H-NMR and 13C-NMR Spectra (see below)

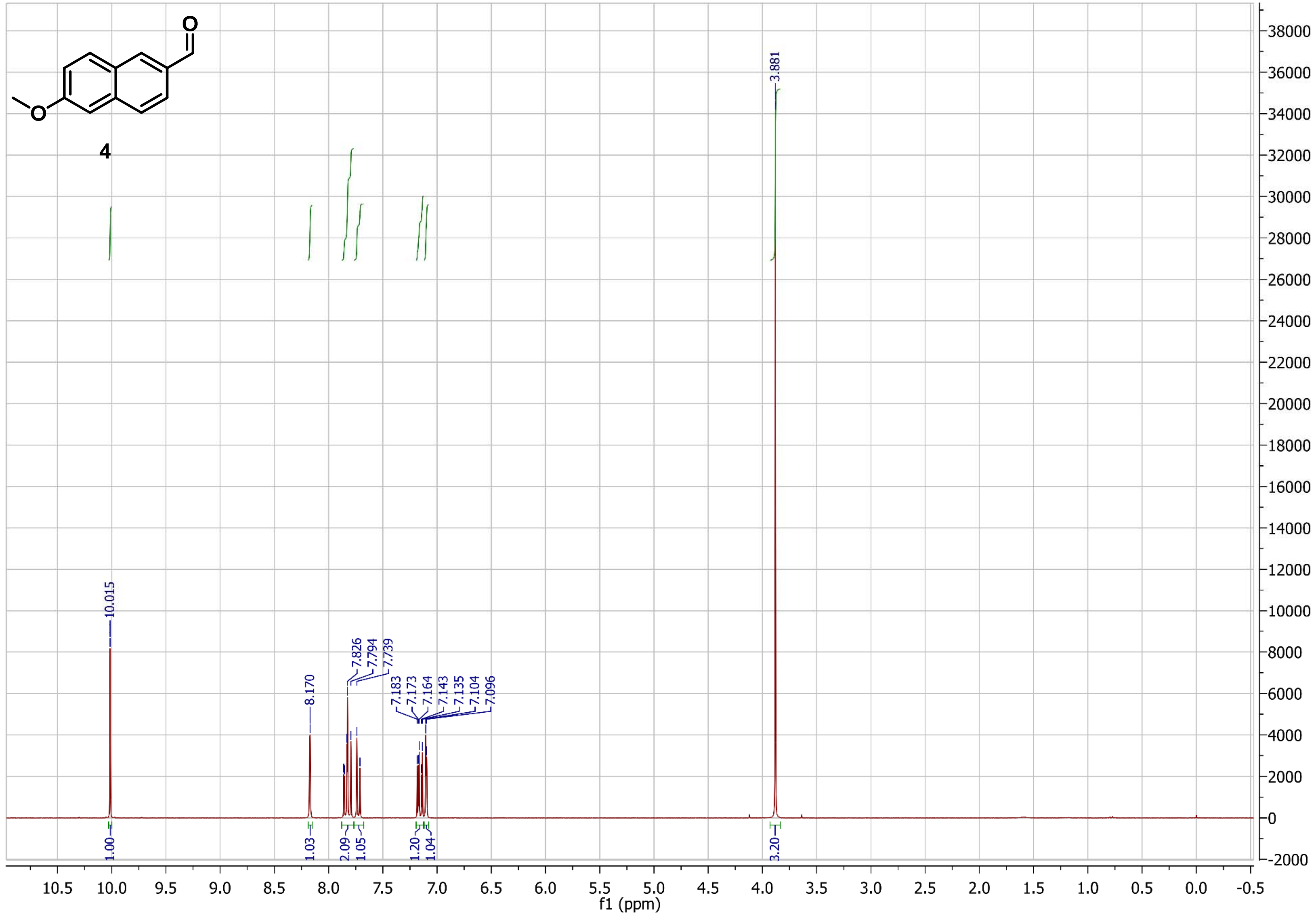

4
10.015
8.170
7.826
7.794
7.739
7.183
7.173
7.164
7.143
7.135
7.104
7.096
3.881
1.00
1.03
2.09
1.05
1.20
1.04
3.20
f1 (ppm)

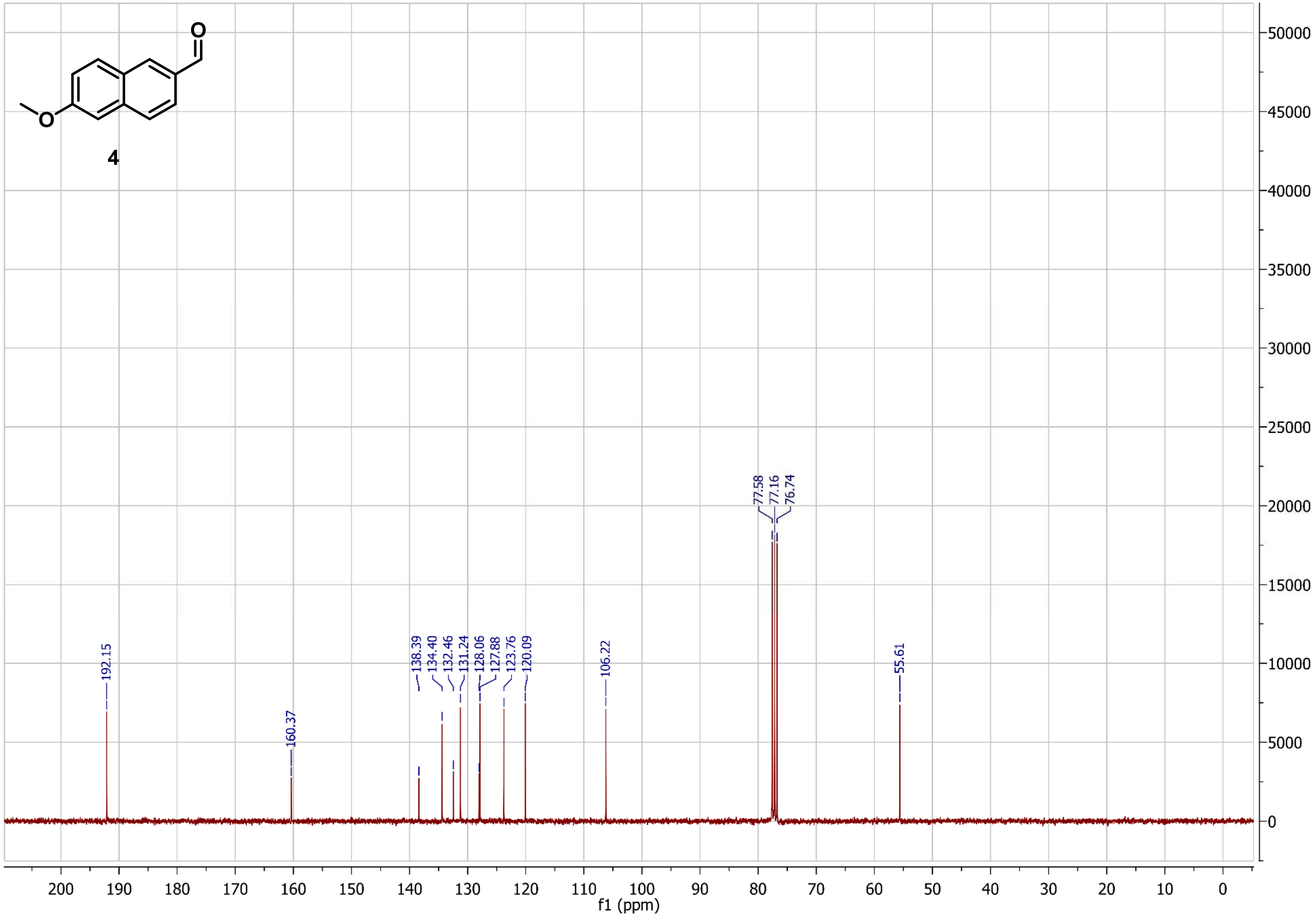
4
192.15
160.37
138.39
134.40
132.46
131.24
128.06
127.88
123.76
120.09
106.22
77.58
77.16
76.74
55.61
f1 (ppm)

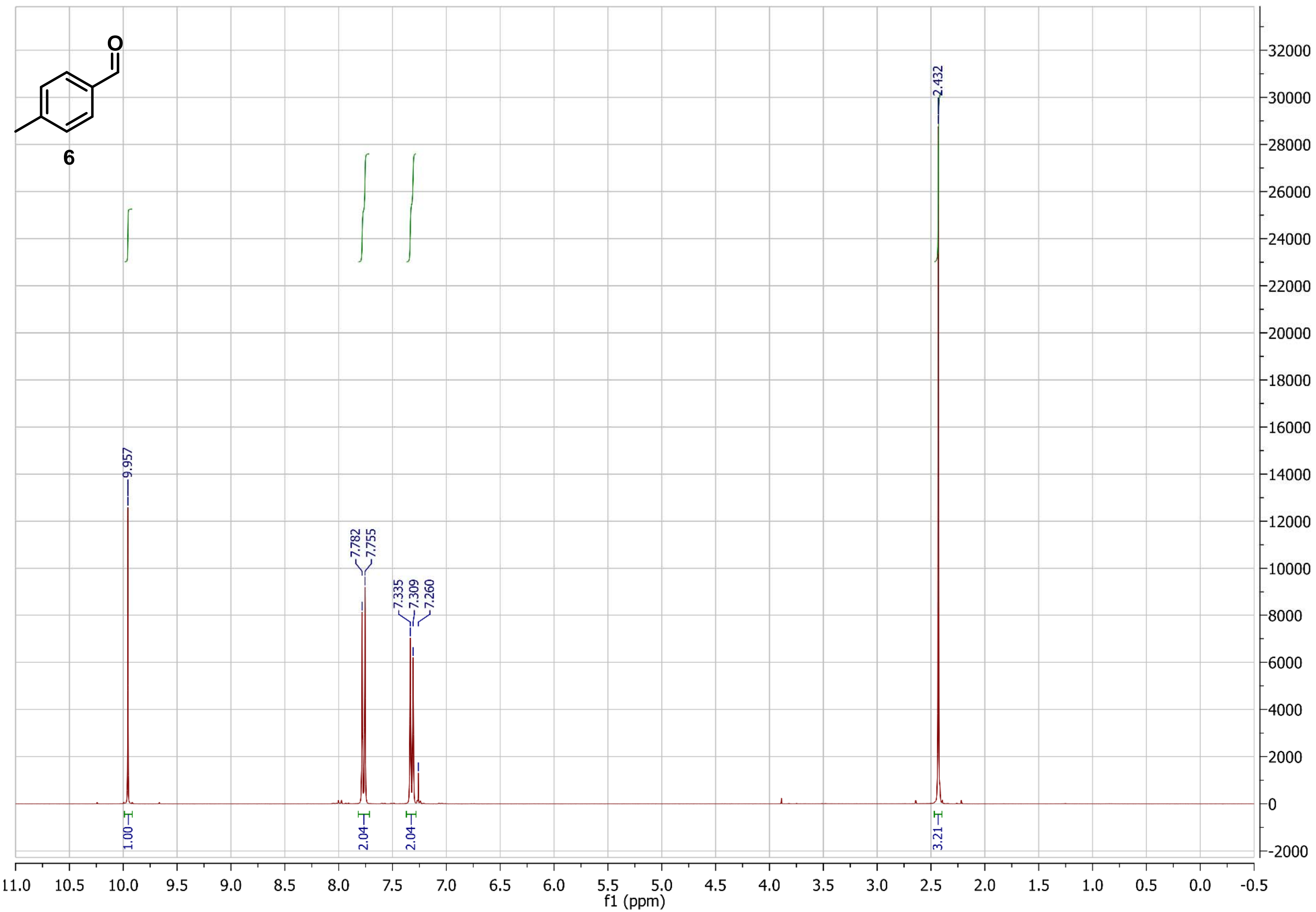

O
6
9.957
7.782
7.755
7.335
7.309
7.260
2.432
1.00
2.04
2.04
3.21
32000
30000
28000
26000
24000
22000
20000
18000
16000
14000
12000
10000
8000
6000
4000
2000
0
-2000
11.0 10.5 10.0 9.5 9.0 8.5 8.0 7.5 7.0 6.5 6.0 5.5 5.0 4.5 4.0 3.5 3.0 2.5 2.0 1.5 1.0 0.5 0.0 -0.5
f1 (ppm)

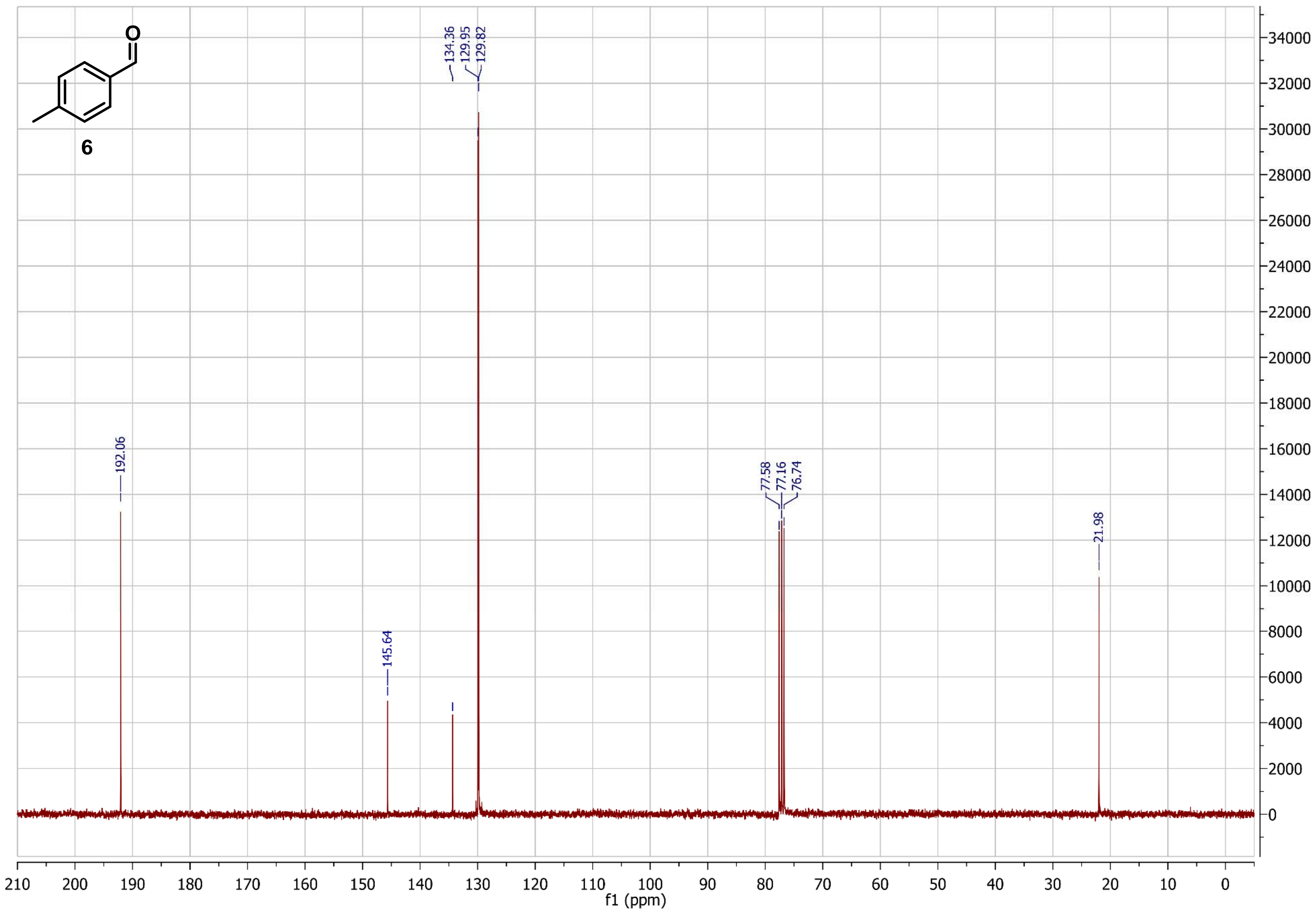

6
134.36
129.95
129.82
192.06
145.64
77.58
77.16
76.74
21.98
f1 (ppm)

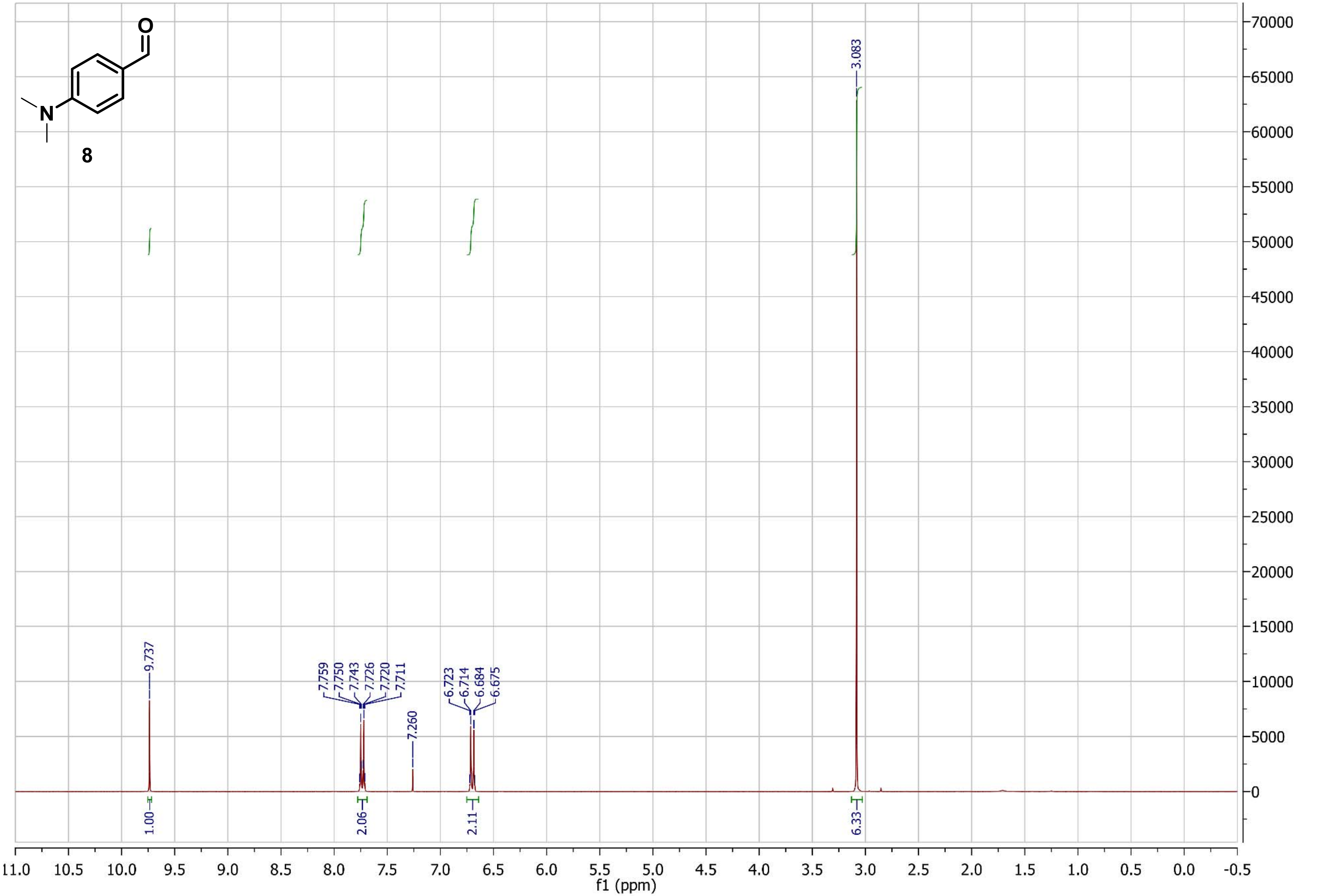

8
9.737
7.759
7.750
7.743
7.726
7.720
7.711
7.260
6.723
6.714
6.684
6.675
3.083
1.00
2.06
2.11
6.33
f1 (ppm)

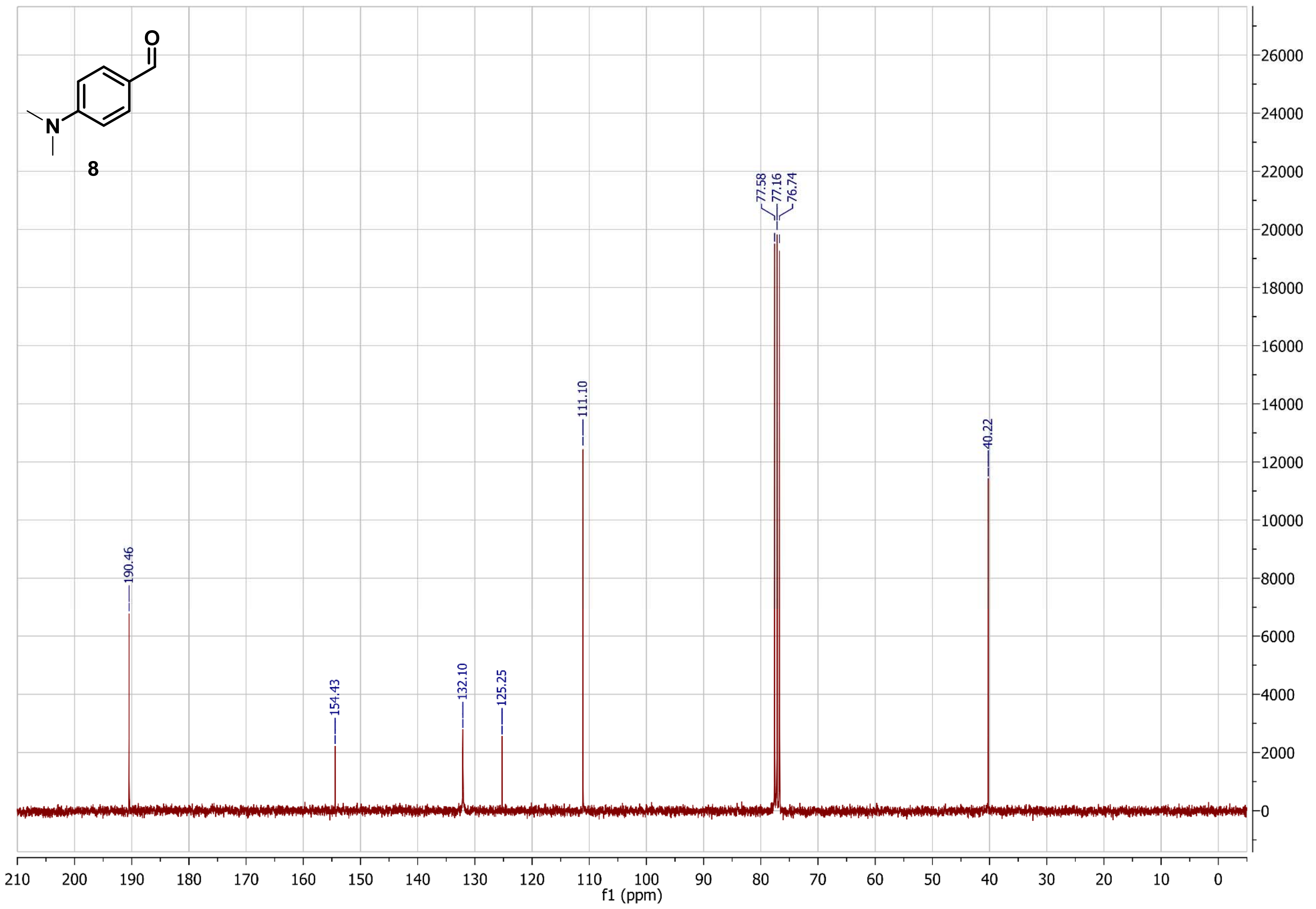

8
190.46
154.43
132.10
125.25
111.10
77.58
77.16
76.74
40.22
f1 (ppm)

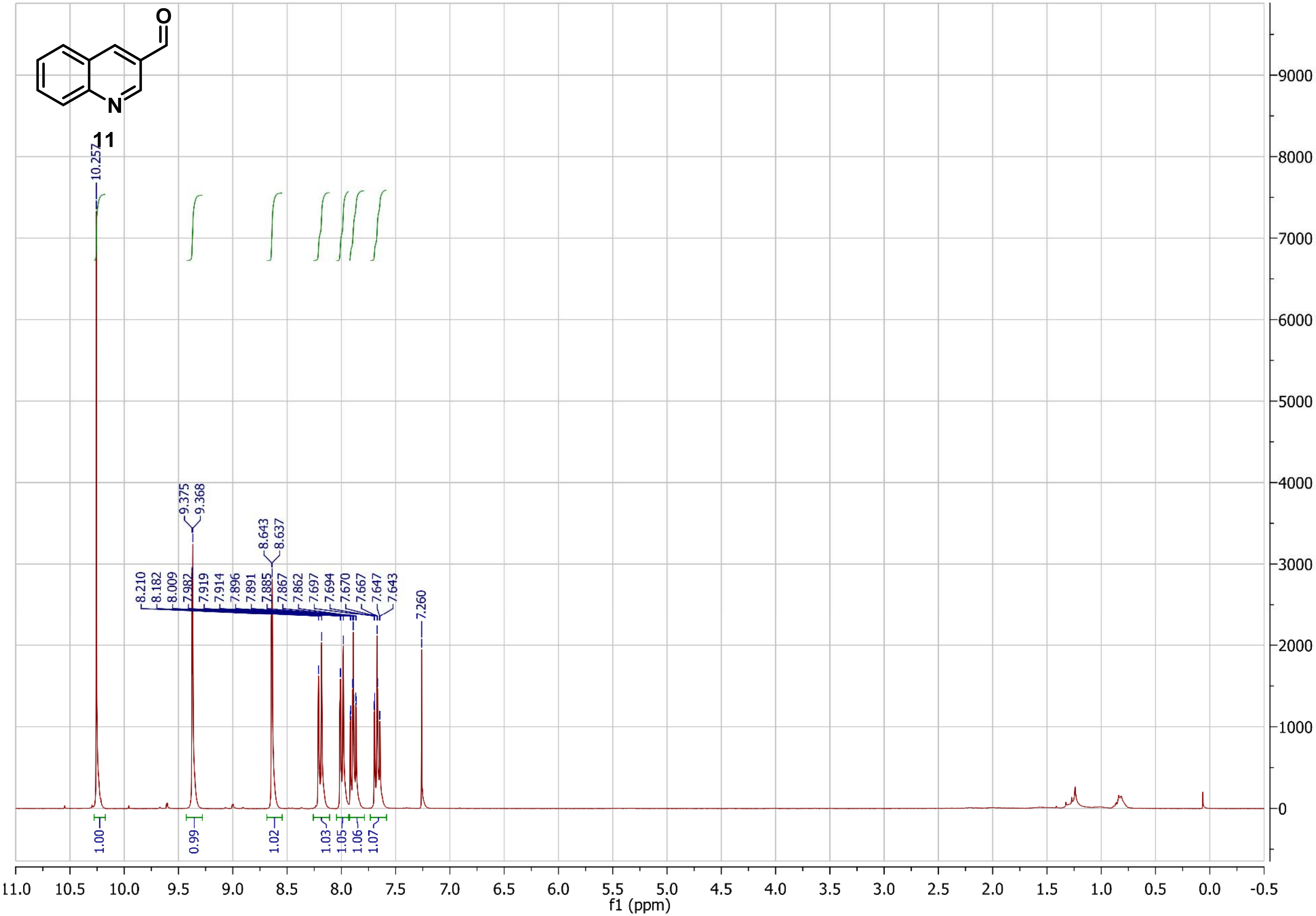

11
10.257
9.375
9.368
8.643
8.637
8.210
8.182
8.009
7.982
7.919
7.914
7.896
7.891
7.885
7.867
7.862
7.697
7.694
7.670
7.667
7.647
7.643
7.260
1.00
0.99
1.02
1.03
1.05
1.06
1.07
f1 (ppm)

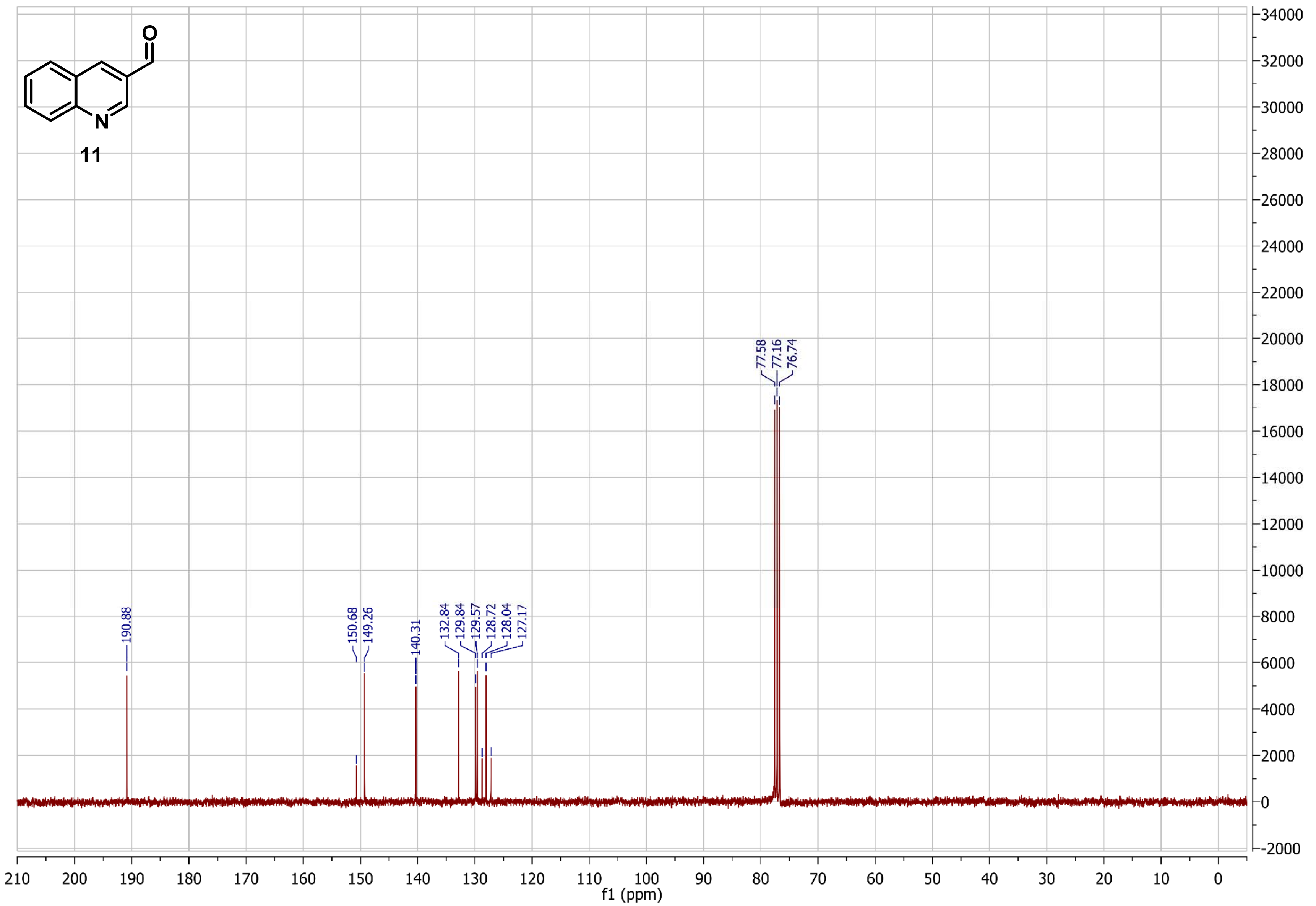
O
N
11
190.88
150.68
149.26
140.31
132.84
129.84
129.57
128.72
128.04
127.17
77.58
77.16
76.74
34000
32000
30000
28000
26000
24000
22000
20000
18000
16000
14000
12000
10000
8000
6000
4000
2000
0
-2000
210 200 190 180 170 160 150 140 130 120 110 100 90 80 70 60 50 40 30 20 10 0
f1 (ppm)

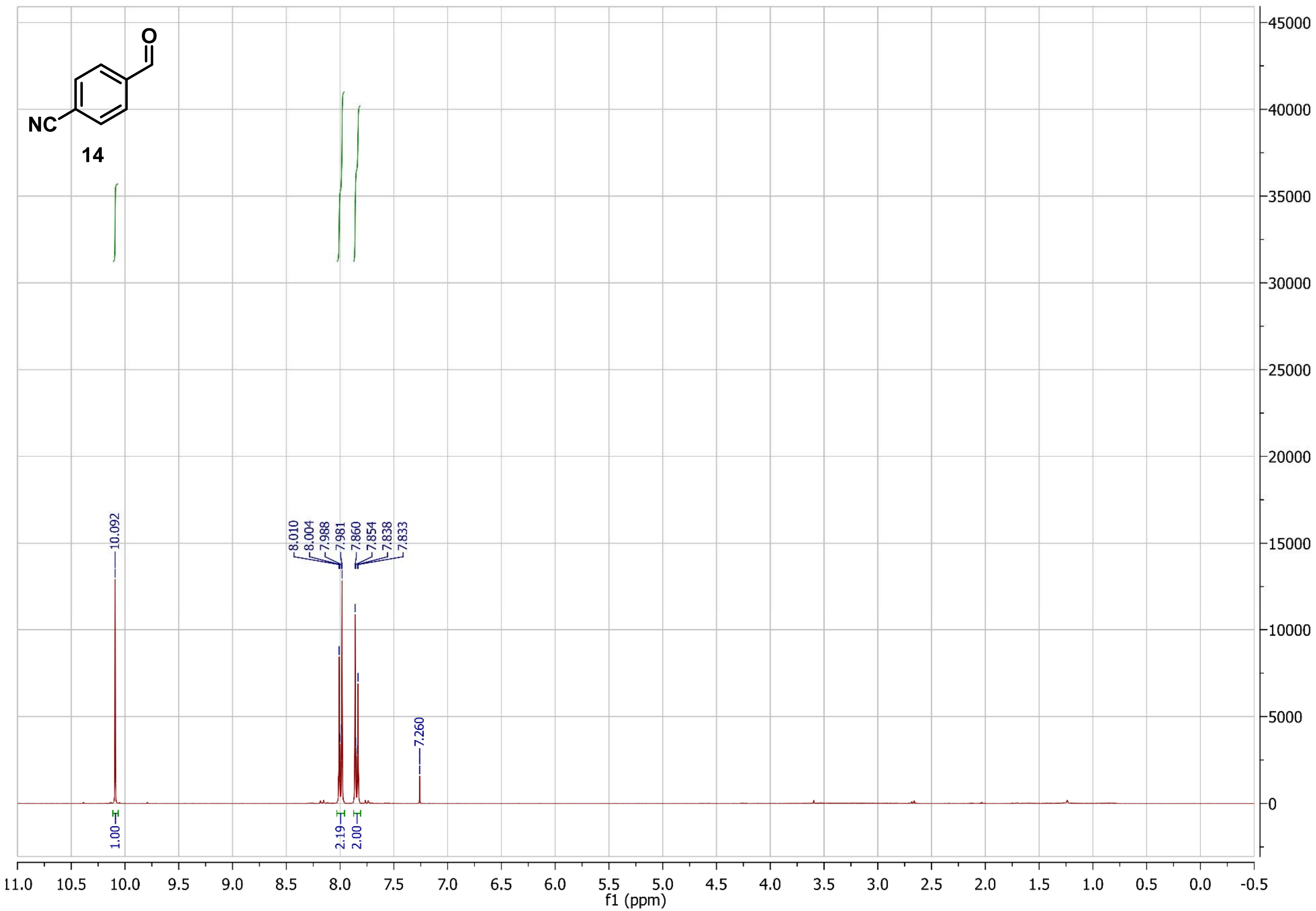
NC
14
10.092
8.010
8.004
7.988
7.981
7.860
7.854
7.838
7.833
7.260
1.00
2.19
2.00
f1 (ppm)

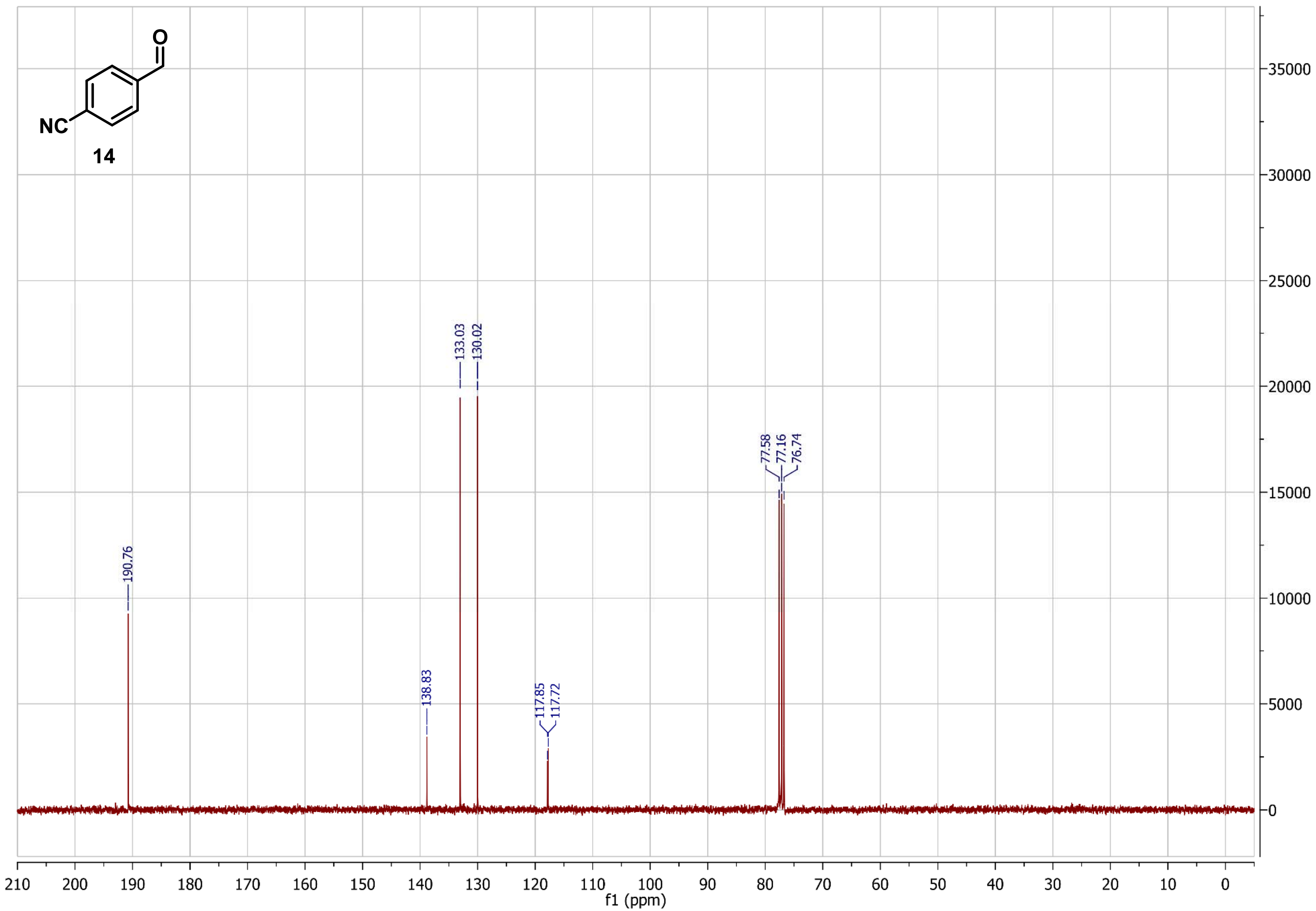
O
NC
14
190.76
138.83
133.03
130.02
117.85
117.72
77.58
77.16
76.74
35000
30000
25000
20000
15000
10000
5000
0
210
200
190
180
170
160
150
140
130
120
110
100
90
80
70
60
50
40
30
20
10
0
f1 (ppm)

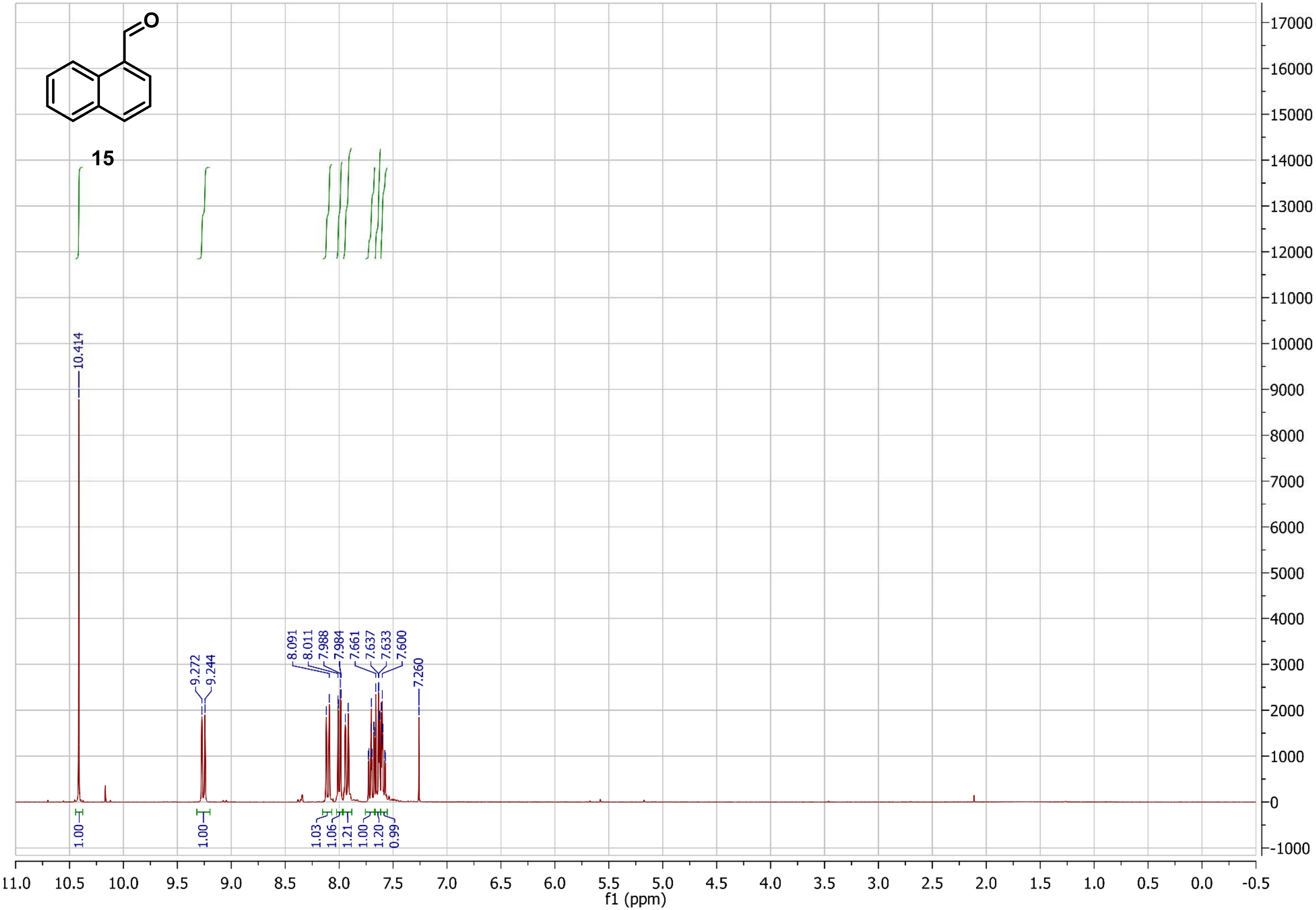
15
10.414
9.272
9.244
8.091
8.011
7.988
7.984
7.661
7.637
7.633
7.600
7.260
1.00
1.00
1.03
1.06
1.21
1.00
1.20
0.99
f1 (ppm)

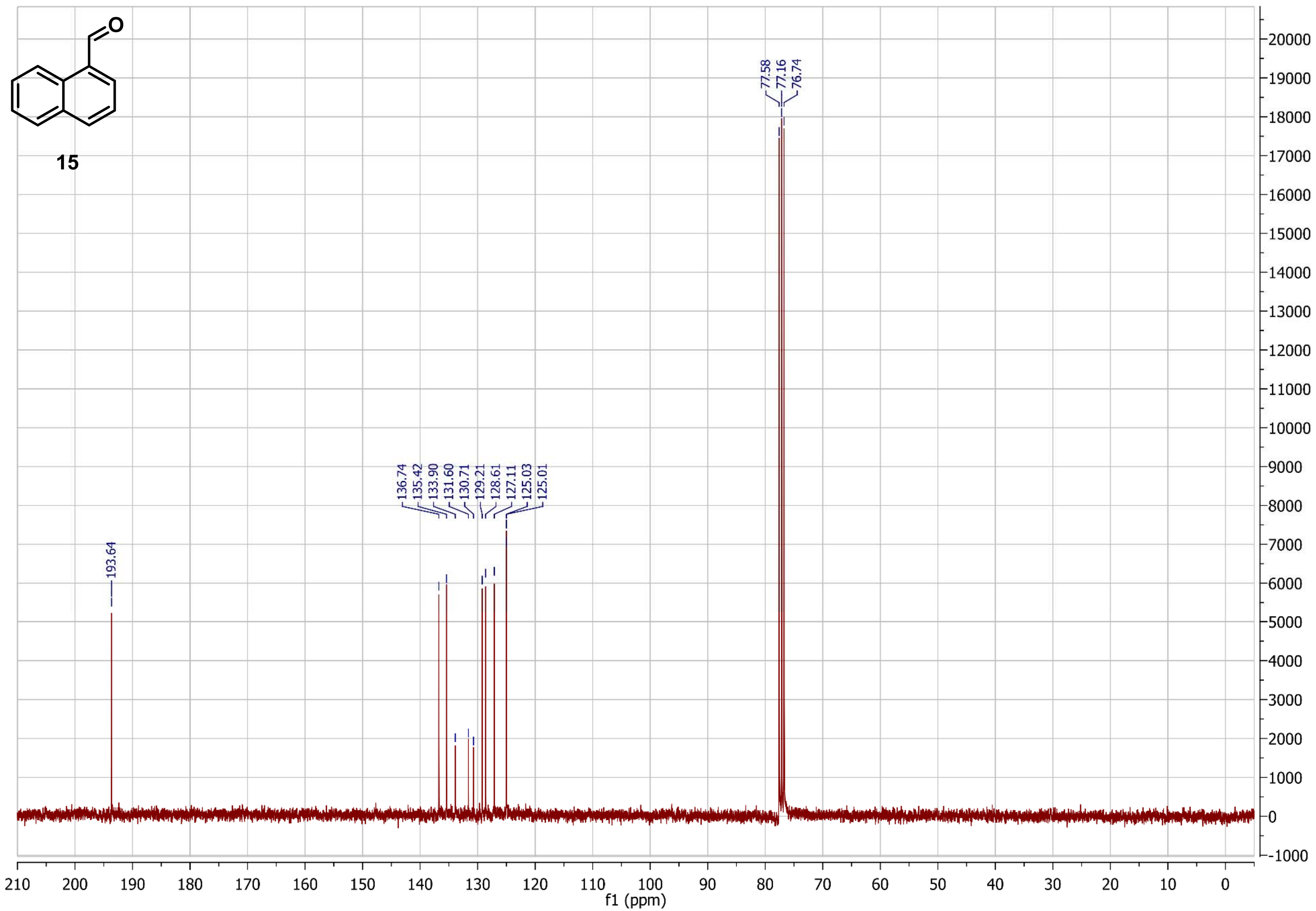
15
193.64
136.74
135.42
133.90
131.60
130.71
129.21
128.61
127.11
125.03
125.01
77.58
77.16
76.74
f1 (ppm)

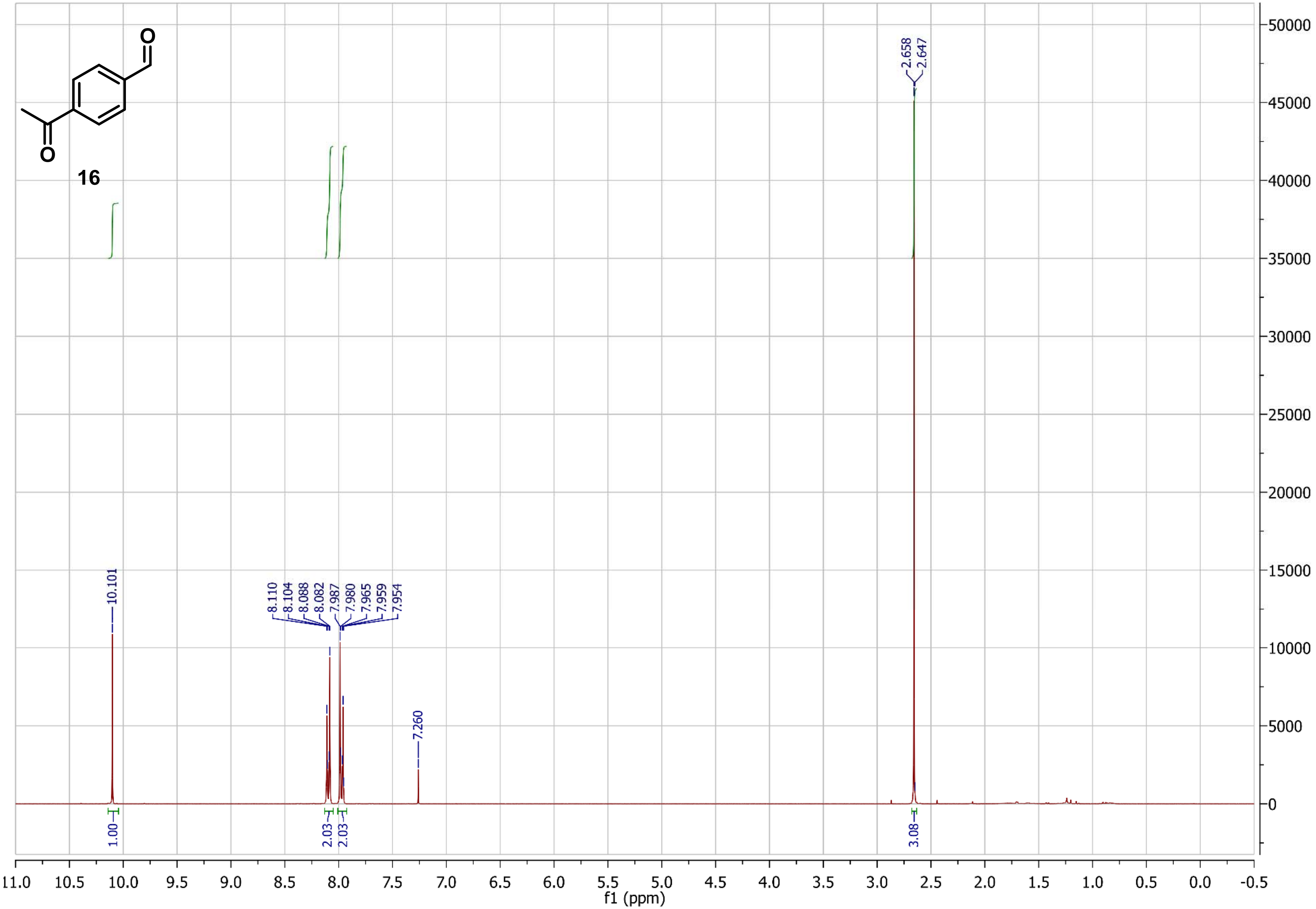

16
10.101
8.110
8.104
8.088
8.082
7.987
7.980
7.965
7.959
7.954
7.260
2.658
2.647
1.00
2.03
2.03
3.08
f1 (ppm)

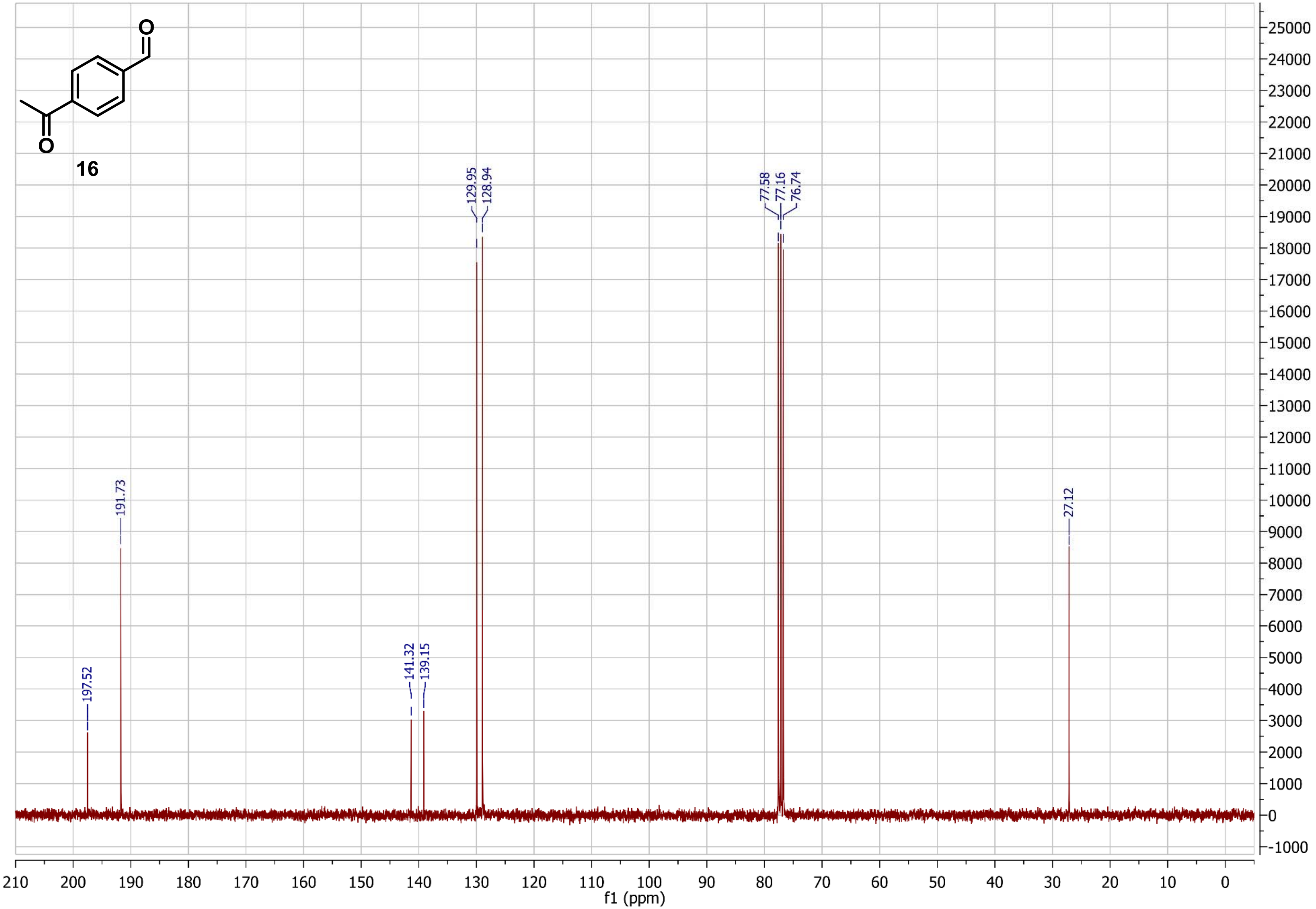
197.52
191.73
141.32
139.15
129.95
128.94
77.58
77.16
76.74
27.12
f1 (ppm)

16

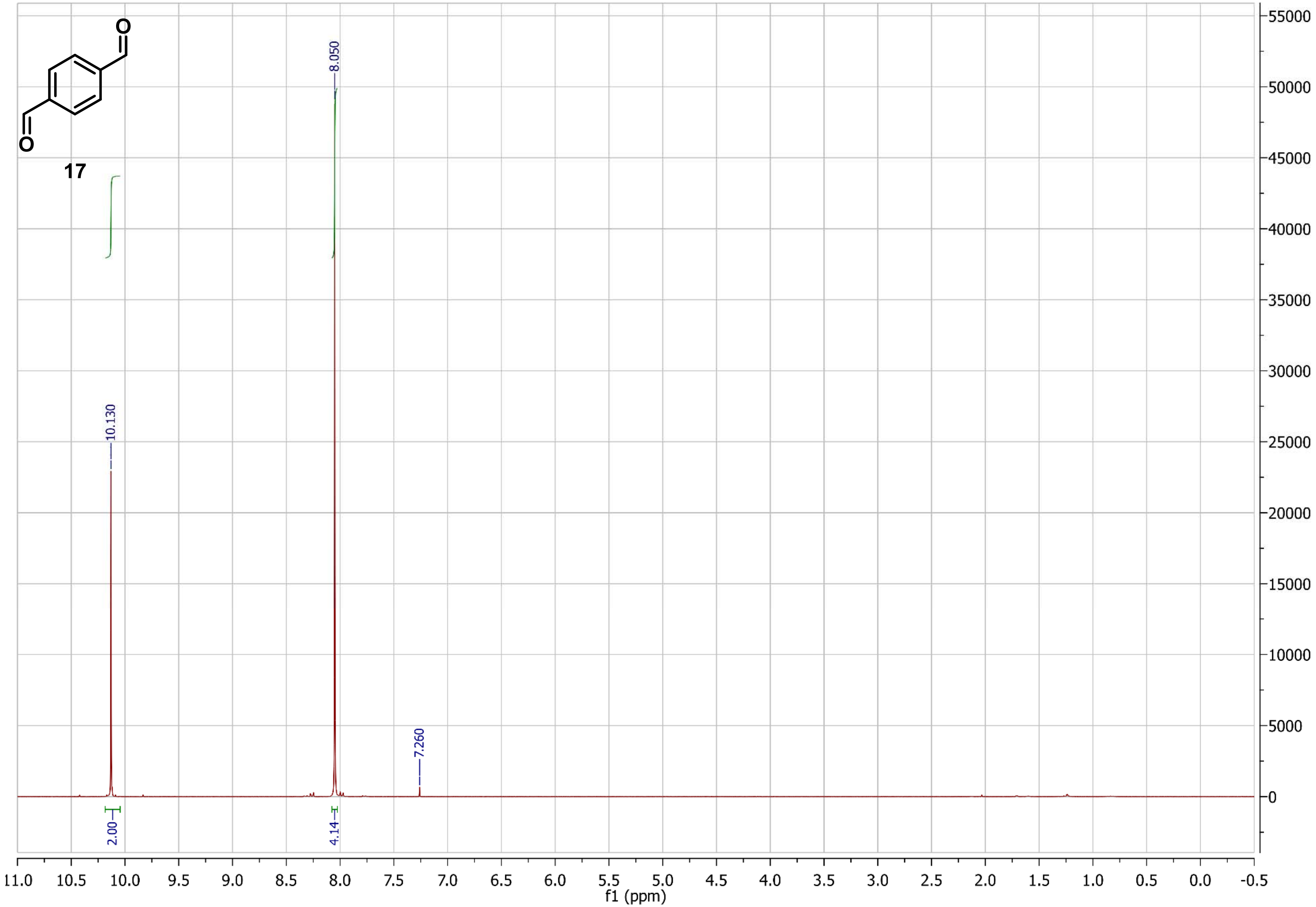
17
8.050
10.130
7.260
2.00
4.14
f1 (ppm)

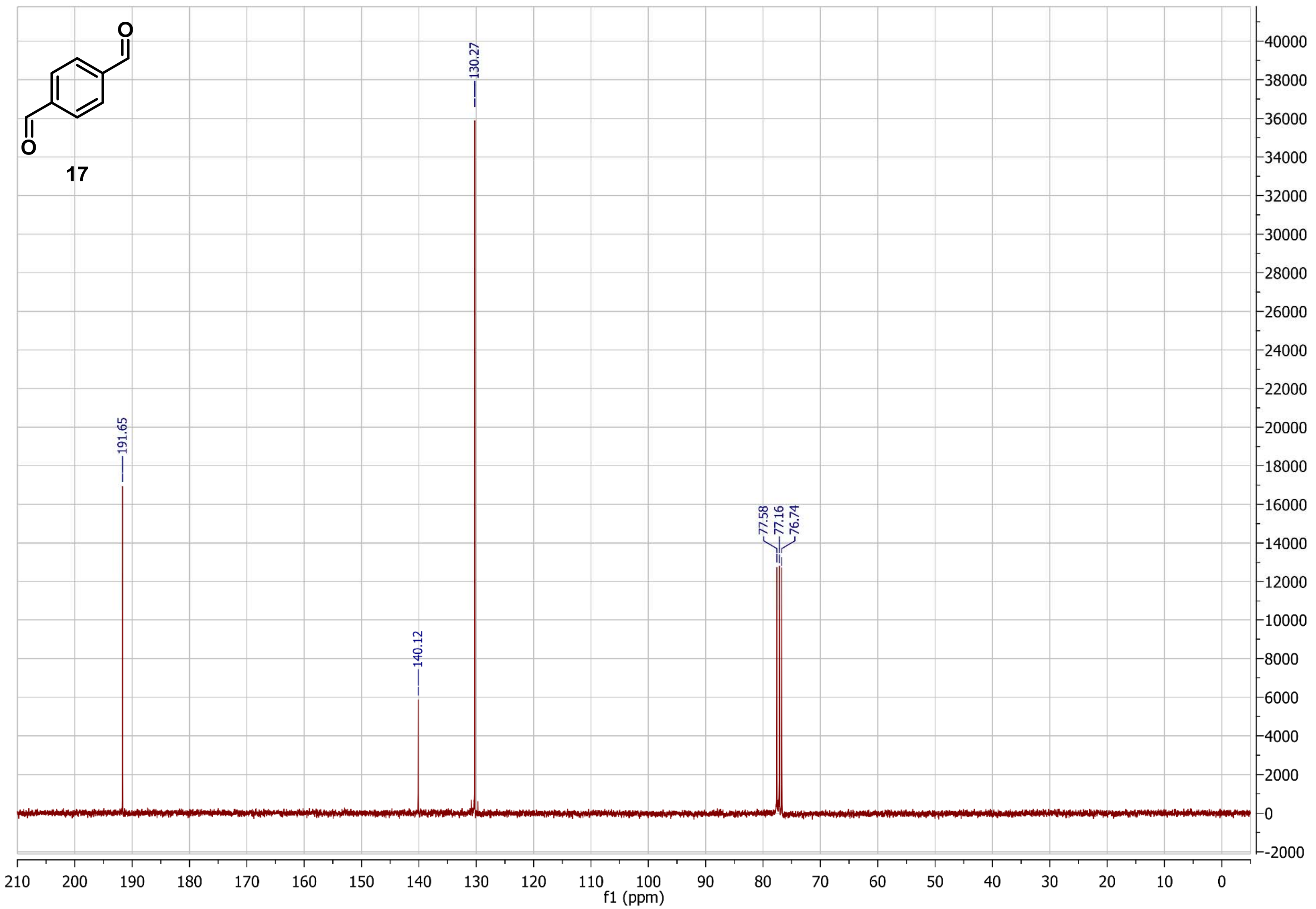
17
191.65
140.12
130.27
77.58
77.16
76.74
f1 (ppm)

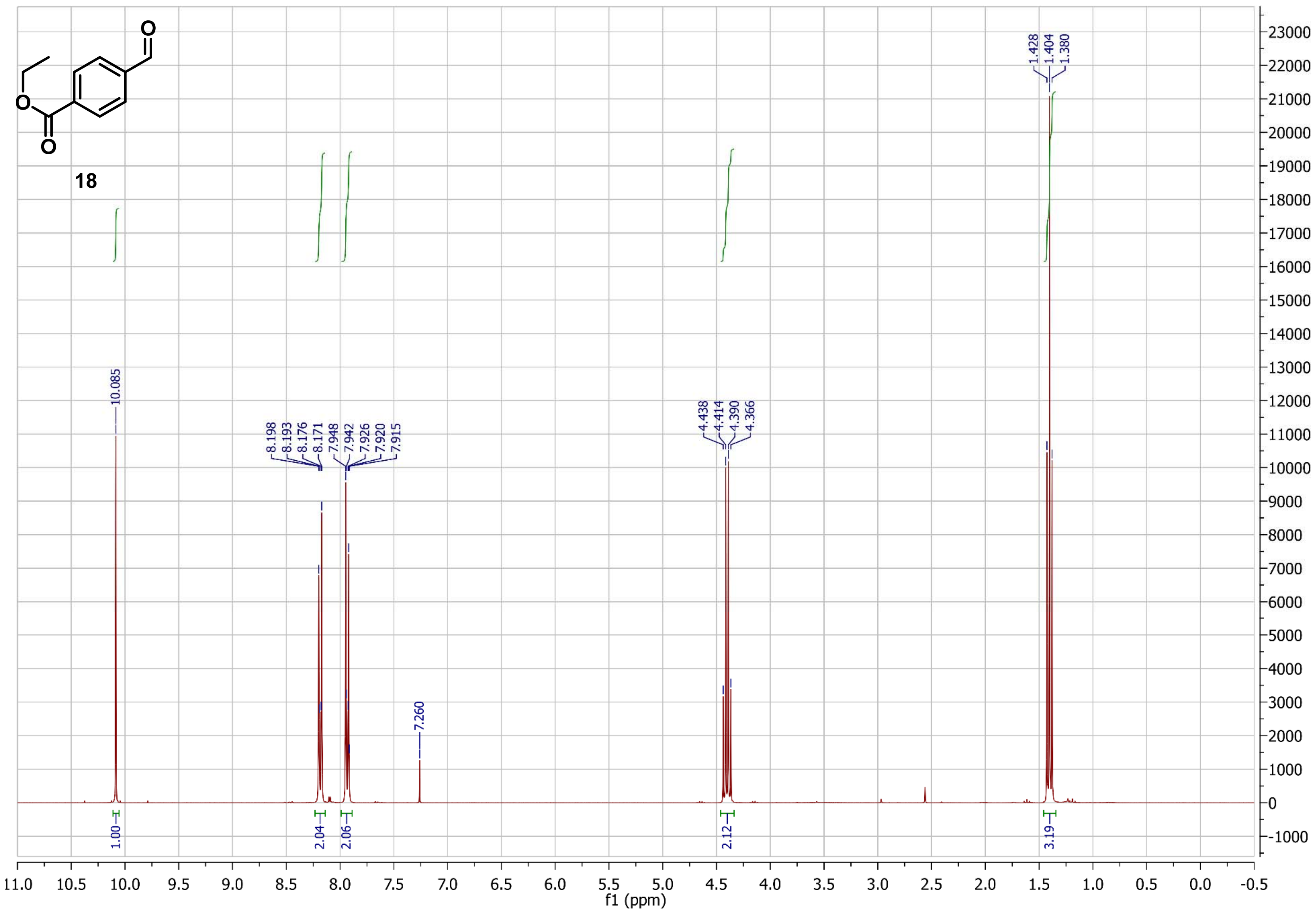

18
10.085
8.198
8.193
8.176
8.171
7.948
7.942
7.926
7.920
7.915
7.260
4.438
4.414
4.390
4.366
1.428
1.404
1.380
1.00
2.04
2.06
2.12
3.19
f1 (ppm)

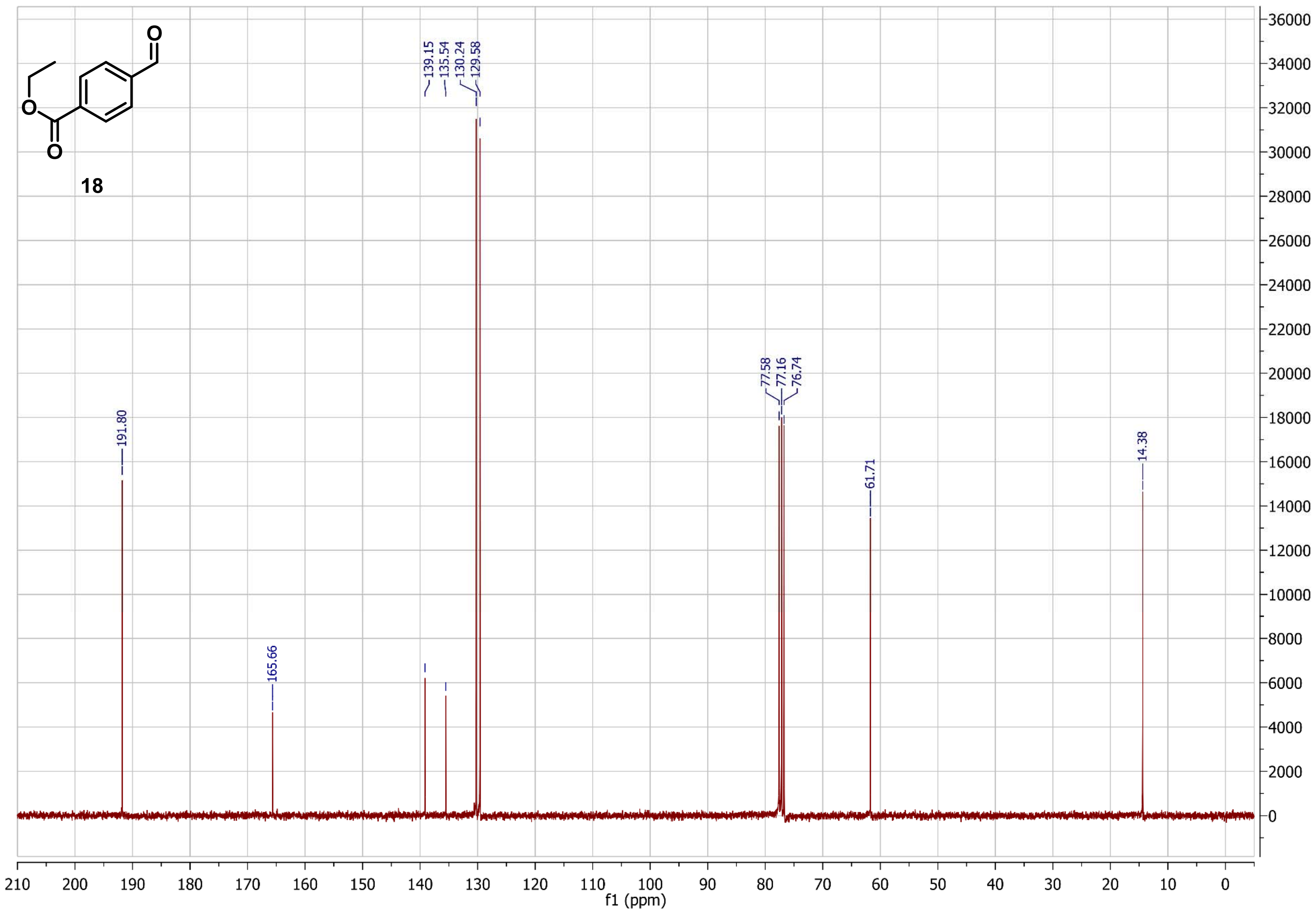

O
O
O
191.80
165.66
139.15
135.54
130.24
129.58
77.58
77.16
76.74
61.71
14.38
f1 (ppm)

18
